# Vertical dynamics of free-living and particle-associated *vibrio* communities in the eastern tropical Indian Ocean

**DOI:** 10.3389/fmicb.2023.1285670

**Published:** 2023-10-19

**Authors:** Shaodong Zhu, Xiaolei Wang, Wenbin Zhao, Yulin Zhang, Derui Song, Haojin Cheng, Xiao-Hua Zhang

**Affiliations:** ^1^Frontiers Science Center for Deep Ocean Multispheres and Earth System, and College of Marine Life Sciences, Ocean University of China, Qingdao, China; ^2^Laboratory for Marine Ecology and Environmental Science, Laoshan Laboratory, Qingdao, China; ^3^Institute of Evolution and Marine Biodiversity, Ocean University of China, Qingdao, China

**Keywords:** vertical dynamics, planktonic vibrios, abundance, community composition, eastern tropical Indian Ocean

## Abstract

Members of the family *Vibrionaceae* (vibrios) are widely distributed in estuarine, offshore, and marginal seas and perform an important ecological role in the marine organic carbon cycle. Nevertheless, there is little knowledge about whether vibrios play ecological roles in the oligotrophic pelagic area, which occupies a larger water volume. In this study, we investigated the abundance, diversity, and composition of free-living and particle-associated vibrios and their relationships with environmental factors along the water depth in the eastern tropical Indian Ocean (ETIO). The abundance of vibrios in free-living fractions was significantly higher than that of particle-associated fractions on the surface. Still, both were similar at the bottom, indicating that vibrios may shift from free-living lifestyles on the surface to mixed lifestyles at the bottom. *Vibrio*-specific 16S rRNA gene amplicon sequencing revealed that *Paraphotobacterium marinum* and *Vibrio rotiferianus* were dominant species in the water column, and *Vibrio parahaemolyticus* (a clinically important pathogen) was recorded in 102 samples of 111 seawater samples in 10 sites, which showed significant difference from the marginal seas. The community composition also shifted, corresponding to different depths in the water column. *Paraphotobacterium marinum* decreased with depth, and *V. rotiferianus* OTU1528 was mainly distributed in deeper water, which significantly correlated with the alteration of environmental factors (e.g., temperature, salinity, and dissolved oxygen). In addition to temperature and salinity, dissolved oxygen (DO) was an important factor that affected the composition and abundance of *Vibrio* communities in the ETIO. Our study revealed the vertical dynamics and preferential lifestyles of vibrios in the ETIO, helping to fill a knowledge gap on their ecological distribution in oligotrophic pelagic areas and fully understanding the response of vibrios in a global warming environment.

## Introduction

The family *Vibrionaceae* (vibrios), belonging to the class *Gammaproteobacteria*, encompasses 12 valid genera and 211 valid species (https://lpsn.dsmz.de/family/vibrionaceae, April 2023). Among them, the genus *Vibrio*, containing most species in the family *Vibrionaceae* (144 species of *Vibrio* were validly published with the correct name), are widely distributed in aquatic environments (including estuaries, open ocean, and sediments) and may play important roles in biogeochemical cycles (Jesser and Noble, 2018; Zhang et al., 2018). Several species of the genus, such as *Vibrio cholerae, Vibrio parahaemolyticus, Vibrio vulnificu*s, *Vibrio anguillarum*, and *Vibrio harveyi* (Oliver and Jones, 2015; Zhang et al., 2020), are well known as pathogenic strains that are capable of infecting humans or aquatic organisms. In particular, *V. cholerae, V. parahaemolyticus*, and *V. vulnificu*s are three clinically important pathogens for humans that pose a significant threat to public health and food safety (Siboni et al., 2016). Most *Vibrio* species are characterized by their halophilic nature, highly plastic genomes, movement using polar flagella, rapid growth, and a broad metabolic range with the capability to use a wide variety of carbon sources (Asplund et al., 2011). In general, *Vibrio* species have comparatively low abundance within the natural microbial community. According to culture-independent methods, the *Vibrio* population in coastal waters is generally <1% of the total bacteria (Thompson et al., 2004), with average abundances ranging between 10^4^ and 10^8^ 16S rRNA gene copies/L in estuarine and coastal waters (Zhang et al., 2018). However, a recent study showed that *Vibrio* abundance increased rapidly, from 4.2 × 10^4^ copies/ml up to 8.1 × 10^5^ copies/ml, with the relative abundance increasing from <1 to 20.54% due to the influence of dissolved organic matter derived from *Ulva prolifera* (Liang et al., 2021). In addition, considering the ability of vibrios to use large amounts of organic carbon compounds (e.g., chitin, alginic acid, and agar) as carbon and energy sources, they may play a more important role in the carbon cycle in marine environments, which was underestimated previously (Takemura et al., 2014; Zhang et al., 2018).

Previous studies on the abundance and diversity of *Vibrio* communities and their response to environmental factors are almost all focused on estuarine, offshore, and marginal seas (Siboni et al., 2016; Zhang et al., 2018; Liang et al., 2019; Wang et al., 2020). Distinct distribution patterns of *Vibrio* communities have been found in different marginal seas, which may be driven by changes in spatial and environmental factors (Siboni et al., 2016; Jesser and Noble, 2018; Wang et al., 2022; Williams et al., 2022). In seawater, community compositions of *Vibrio* spp. were distinguished by sampling area, with different dominant groups in the Bohai Sea (*Vibrio caribbeanicus*), Yellow Sea (*Vibrio chagasii* and *V. harveyi*), East China Sea, and South China Sea (*V. japonicus* and *V. chagasii*) (Liang et al., 2019; Wang et al., 2022). Vibrios in sediments of Chinese marginal seas also varied significantly among sampling areas (Wang et al., 2019). To the best of our knowledge, there are no reports regarding the ecological distribution of total vibrios in the open ocean (Zhang et al., 2018). Compared with marginal seas, open oceans cover a much larger area and occupy a larger water volume, which suggests that the *Vibrio* community may play a greater ecological role. Therefore, to fully understand the roles of vibrios in the global marine carbon cycle, it is essential to study the distribution patterns of vibrios in the open ocean. Based on cultivation methods, it has been reported that the *Vibrio* species can survive in the open ocean. Ten *Vibrio* species have been isolated from seawater at different depths of the Western Pacific (Sun et al., 2021). They occupied a high proportion in the cultivable bacterial community at 0–6,000 m seawater from the Mariana Trench (Zhao et al., 2020). However, culture methods alone cannot reflect the total community distribution of vibrios in environments. Therefore, it is necessary to investigate the distribution patterns of total *Vibrio* spp. based on more reliable methods, such as high-throughput sequencing technologies (Sogin et al., 2006). The ecological significance of the *Vibrio* community in the open ocean needs to be further effectively evaluated.

*Vibrio* populations exhibit either of two alternative growth strategies—free-living or associated with marine particles and/or living hosts to acquire nutrients or to avoid predators (Matz et al., 2005; Thompson and Polz, 2006; Asplund et al., 2011). In the Northern Chinese Marginal Seas, the abundance of *Vibrio* spp. determined by qPCR revealed that free-living (FL) communities are significantly higher than those of particle-associated (PA) communities (Liang et al., 2019). It has also been reported that the growth strategies of the *Vibrio* community switch along with the change in surrounding environments. It has been reported that the alteration of *Vibrio* lifestyle is affected by genotypes (Takemura et al., 2014). For instance, *V. cholerae* (N16961) proliferates and shifts from being free-living cells to attached cells under conditions where particle formation occurs in the dying phytoplankton-bloom (*Pyramimonas*) (Worden et al., 2006). Meanwhile, various environmental factors, such as salinity, temperature, and chlorophyll *a*, have been demonstrated to affect *Vibrio* lifestyles (Takemura et al., 2014). The determining environmental factors and optimum growth conditions vary across the *Vibrio* species (Takemura et al., 2014), and the succession of the *Vibrio* community in response to the change in environmental factors varies across the study sites. For instance, a potential transition of preferential lifestyles of *Vibrio* spp. from particle-associated in the Yellow Sea (China) to free-living in the East China Sea and South China Sea (Wang et al., 2022) has been reported. Additionally, the lifestyle of vibrios has been observed at a particular time or in a particular sea area, such as the northern Chinese marginal seas, the Sansha Yongle Blue Hole, and the Baltic and Skagerrak Seas (Unanue et al., 1992; Eiler et al., 2006; Liang et al., 2019; Li et al., 2020). These studies jointly reveal that the particle-associated mode is the major lifestyle of *Vibrio* spp., and free-living may serve as a temporary state. Thus, in the eastern tropical Indian Ocean (ETIO), where the nutrient usually shows more limitations than the marginal seas, the change of vibrios lifestyle from the surface to the bottom seawater needs to be further studied.

The ETIO is primarily dominated by the Indian monsoon, which is separated by two distinct periods—the Southwest monsoon from June to September and the Northeast monsoon from December to March (Fazeli et al., 2013). Due to the presence of the warm pool in the ETIO, it is characterized by very high sea surface temperatures (SST; >28°C) (Xuan et al., 2015). There is a strong vertical temperature gradient located at depths of 50–150 m, indicating the presence of a strong thermocline below the mixed layer (Xuan et al., 2015). Influenced by an uneven spreading of freshwater from the interior of the Bay of Bengal (BOB), the low-saline water appears near the sea surface in the bay mouth (~6°N) (Jensen, 2003; Sengupta et al., 2006). In the BOB, located in the northern of the ETIO, the dissolved oxygen concentration in 100–1,000 m of water is very low, forming an oxygen minimum zone in this position (Bertagnolli and Stewart, 2018; Sridevi and Sarma, 2020). Additionally, quite low concentrations of dissolved inorganic nutrients suggest the ETIO is a typical oligotrophic ocean (Wu et al., 2019). Strong stratification, warm surface water, uneven salinity, regional low-oxygen water layer, and typical oligotrophic conditions make the ETIO a very different environment from marginal seas and maintain various microbes. The vertical stratification of microbial communities in ETIO was identified in total bacteria, anammox bacteria, and diazotrophs (Wang et al., 2016; Qian et al., 2018; Wu et al., 2019). It was found that bacterial community structures in the ETIO exhibited a more pronounced stratified distribution pattern in the upper 150 m (Wang et al., 2016). As heterotrophic bacteria, do *Vibrio* also exhibit a similar distribution pattern in the ETIO water column? Wu et al. detected *Vibrio diazotrophicus* as diazotrophs in waters above 200 m based on *nifH* gene amplification and Illumina sequencing in the ETIO (Wu et al., 2019). *Vibrio* communities across a pollution gradient in the Karnaphuli estuary were studied to unravel their biogeochemical drivers (Kopprio et al., 2020). However, the vertical dynamics and the environmental responses of *Vibrio* communities from surface to bottom seawater in the ETIO are still unexplored. The Threshold Indicator Taxa Analysis (TITAN) is a sensitive and precise method to identify ecological community thresholds (Baker and King, 2010) that can detect changes in taxa distributions along an environmental gradient over space or time (Liu et al., 2022). Therefore, the application of TITAN can help to effectively understand the responses of *Vibrio* species to different environmental factors.

In the present study, we hypothesize that the distribution patterns of the *Vibrio* community exhibit continuous differences corresponding to the environmental factors from surface to bottom seawater in the ETIO. To explore the preferential lifestyles of *Vibrio*, the abundance and community structure, as well as the respective influencing factors for the FL and PA *Vibrio* communities, were investigated. The *Vibrio* abundances were determined by qPCR, and community components were analyzed according to the data from high-throughput sequencing. The abundances and compositions of *Vibrio* spp. correlated with a range of environmental factors. The effects of large temperature spans along varying water depths (from the surface to the bottom) on *Vibrio* diversity in the ETIO may help us reveal the response of vibrios to a global warming environment and reduce the potential risk of pathogenic *Vibrio* outbreaks.

## Materials and methods

### Study site and sample collection

**Figure 1:**
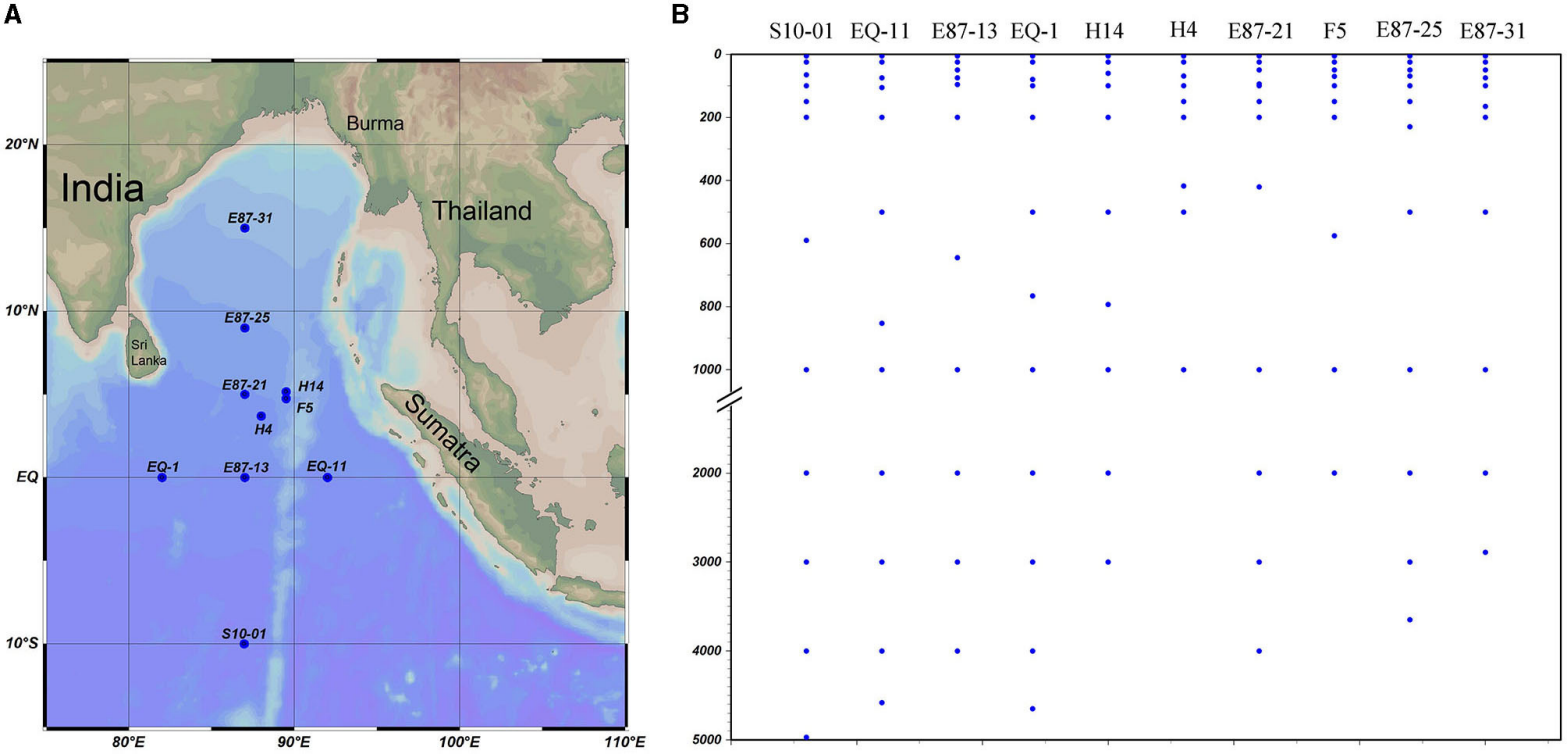
Site description and sampling profile in the vertical direction (the map was created using Ocean Data View [version 5.5.2; R. Schlitzer, Ocean Data View, https://odv.awi.de, 2021]). Study area and sampling stations **(A)** and sampling profile at vertical direction **(B)**.

Samplings were conducted from the eastern tropical Indian Ocean (ETIO) onboard the *Shiyan 3* during a summer cruise (29 April to 5 June 2021). A total of 111 seawater samples from 10 vertical sites (S10-01, EQ-11, E87-13, EQ-1, H14, H4, E87-21, F5, E87-25, and E87-31) were collected using a Sealogger conductivity-temperature-depth (CTD; SBE 911PlusCTD) rosette water sampler (Figure 1). Physicochemical parameters, including water depth, temperature, salinity, and dissolved oxygen (DO), were recorded by a Seabird 911Plus CTD.

Approximately 1 L of seawater was filtered through 3 μm and 0.22 μm polycarbonate membranes (GTTP, 47 mm, Ispore) using a vacuum pump under low, non-disruptive pressure (<5 mm Hg). The microorganisms on the 3-μm filter were considered bacteria associated with smaller particles and algae (Teeling et al., 2012), and the microorganisms on the 0.22-μm filter were considered free-living bacteria in seawater (Liu et al., 2015). All filters were immediately frozen and stored in liquid nitrogen onboard and transferred to a −80°C freezer in the laboratory until DNA extraction. Samples for nutrients were collected, and the nutrients in each sample were measured based on the classical colorimetric method (Grasshoff et al., 1999), where ${\text{NO}}_{2}^{-}$, ${\text{NO}}_{3}^{-}$, ${\text{NH}}_{4}^{+}$, dissolved silicon (DSi), and dissolved inorganic phosphorus (DIP) were measured by copper-cadmium column reduction method, indophenol blue method, silico-molybdate complex method, and phosphor-molybdate complex method. Water samples (500 ml) for Chlorophyll *a* (Chl *a*) analysis were filtered through a GF/F filter using a vacuum pump (<10 mmHg). Then, the filters were wrapped in aluminum foil and stored in the dark at −20°C. They were extracted with 90% acetone and kept in the dark at 4°C for 24 h, after which Chl *a* concentrations were determined by a Turner Designs Trilogy fluorometer (Parsons et al., 1984).

### DNA extraction and quantitative PCR

DNA was extracted from the 3-μm and 0.22-μm polycarbonate membranes according to the method described by Yin et al. (2013) with some modifications. Each polycarbonate membrane that seawater was filtered through was cut into pieces under sterilized conditions and transferred into an asepsis pipe with 500 μl of sodium chloride-Tris-EDTA (STE) buffer. The mixture was violently shaken on a FastPrep-24 homogenization system (MP Biomedicals, Irvine, California, USA) two times to facilitate cell lysis at the speed of 6.0 m/s. Then, the DNA was extracted by a DNeasy PowerWater Kit (QIAGEN, United States) according to the operation instructions. DNA quantity and purity were evaluated using a Nanodrop-2000 Spectrophotometer (ND-2000; Thermo Fisher Scientific). The extracted DNA was preserved at −80°C until used.

In order to quantify the abundance in the *Vibrio* community, 16S rRNA gene-targeted qPCR was performed. Each DNA sample was measured by a QuantStudio™ 5 System (Applied Biosystems) and QuantStudio™ Design and Analysis Software. V567F and V680R, the specific 16S rRNA oligonucleotide primers for genus *Vibrio*, were used in qPCR with SYBR-green detection (Table 1) (Thompson et al., 2004; Vezzulli et al., 2012). According to the methods from Wang et al. (2022), the 16S rRNA genes of *Vibrio rotiferianus* WXL191 (our laboratory) were selected as nucleic acid templates to prepare the standards. Standard curves were run with every plate, and ddH_2_O instead of template DNA was added as a no-template control (NTC). All extracted DNA were tested in triplicate. All amplification efficiencies of qPCR were always between 95 and 105%, with *R*^2^ values of >0.99.

**Table 1:** Vibrionic oligonucleotide primers for qPCR amplification and sequencing.

**Primers**	**Sequences (5^′^-3^′^)**	**Information on target gene**	**References**
V567F/V680R	GGCGTAAAGCGCATGCAGGT /GAAATTCTACCCCCCTCTACAG	General *Vibrio* spp. (113 bp)	Thompson et al., 2004; Vezzulli et al., 2012
V169F/V680R	GGATAACCTATTGGAAACGATG /GAAATTCTACCCCCCTCTACAG	General *Vibrio* spp. (511 bp)	Siboni et al., 2016

### High-throughput sequencing and *Vibrio* diversity

To determine the overall composition of the *Vibrio* community, the hypervariable regions of V2–V4 targeted by the *Vibrio*-specific primers V169F and V680R (Siboni et al., 2016) in the 16S rRNA gene were amplified (Table 1). Positive amplicons were confirmed by agarose gel electrophoresis. After the PCR products were purified from 2% agarose gels using AxyPrep DNA gel extraction kit (Axygen Biosciences, Union City, CA) and further quantified by QuantiFluor-ST (Promega) according to the manufacturer's protocol, the amplicons were pooled in equimolar and paired-end sequenced (2 × 300) on an Illumina Miseq PE300 platform (Illumina) at Majorbio Bio-Pharm Technology. After raw fastq files were joined using FLASH (Gyraite et al., 2019), operational taxonomic units (OTUs) clustering at a 97% sequence similarity level was also performed with UPARSE (version 11), and chimeric sequences were ascertained and dislodged using UCHIME software (Edgar et al., 2011). The taxonomy of each representative OTU 16S rRNA gene sequence was assigned using the RDP Classifier (Wang et al., 2007) against the SILVA 138 16S rRNA database (http://www.arb-silva.de) with a minimum confidence threshold of 70%. The *Vibrio* sequences were reassigned against the EzBioCloud database (https://www.ezbiocloud.net/) to acquire a more accurate taxonomic identification. To eliminate the effect of sampling effort on analysis, sequences were subsampled according to the minimum number of sample sequences for all samples with a ‘single rarefaction' QIIME script (Caporaso et al., 2010).

### Statistical analysis

The differences in environmental parameters and qPCR data between sampling sites and depth layers were tested using the chi-squared and Kruskal-Wallis tests. The correlations between the *Vibrio* abundance and environmental parameters were determined using Spearman's rank correlation analysis package. All the above statistical analyses were operated using IBM SPSS Statistics version 24.0.0.2 (StatSoft, Tulsa, OK, USA).

The alpha diversity indices, including Good's coverage, Shannon index, Shannon evenness, Chao 1, and phylogenetic distance, were calculated using MOTHUR software packages (Schloss et al., 2009). For beta diversity analysis, non-metric multidimensional scaling (NMDS) based Bray-Curtis dissimilarity was performed at the OTU level with the vegan package v2.6-4 (Oksanen et al., 2022) of the R software v4.2.0. Redundancy analysis (RDA) or canonical correspondence analysis (CCA) was also conducted to determine the relationship between *Vibrio* composition and environmental factors in R software based on the OTU level. ${\text{NO}}_{2}^{-}$, ${\text{NO}}_{3}^{-}$, ${\text{NH}}_{4}^{+}$, DSi, and DIP data were missing in some samples, and these parameters were removed from the RDA/CCA analysis. Correlations between the percentage composition of taxa and environmental factors were predicated based on the Spearman rank correlation coefficient. Using all OTUs occurring in at least three samples, TITAN (TITAN2 v2.4.1) was conducted to identify indicator species with positive and negative responses to temperature and DO and calculated the threshold values of those indicator species (Baker and King, 2010). Co-occurrence networks were constructed for all samples of FL and PA groups based on OTUs with a sequence number of >20. R packages igraph v1.3.5 (Csardi and Nepusz, 2006), psych v2.2.9 (Revelle, 2021), and Hmisc v4.7-1 (Harrell and Dupont, 2022) were used to calculate the network topological data and Gephi (https://gephi.org) was used for network visualization. Only OTUs with a sequence number of >20 in each group were included to reduce complexity. The Spearman's correlation between OTUs was considered a valid relationship if the *r* was > |0.6| and the *p*-value was < |0.01|. Network topological parameters such as the number of nodes, number of edges, mean node degree, clustering coefficient, average path length, modularity, density, and diameter were calculated.

### Data availability

The Illumina sequencing raw data were deposited in the NCBI Sequence Read Archive (SRA) under accession number SRP422930 (Bioproject accession number PRJNA934765).

## Results

### Environmental parameters

The environmental parameters of seawater collected from 10 sites in the ETIO were measured (Supplementary Table S1). The temperature gradually decreased with depth from the surface water (28–31°C) to deep water and dropped sharply from 50 to 150 m along a vertical gradient. Sea surface salinity ranged from 31.9 (E87-31) to 34.6 PSU (EQ-1). However, the salinity was low in surface water and increased rapidly until the maximum in the subsurface, and then, it decreased slowly with depth and maintained stability below 2,000 m. In addition, the concentration of DO dropped rapidly with depth—from 50 to 200 m—and recovered in depths from 1.000 to 2.000 m. The DO concentration was low, ranging from 200 to 1.000 m, which was consistent with previous observations (Sridevi and Sarma, 2020). In particular, the lowest concentration of DO was noted in station E87-31, which is located in the BOB. According to environmental parameters measured in this study, Chl *a* was distributed in the euphotic layer (200 m above). The Chl *a* maximum layer varied across stations, ranging from 60 to 105 m in depth, and the maximum Chl *a* concentration was 0.770 μg/L at a depth of 94 m in site E87-21. The concentrations of DIP and ${\text{NO}}_{3}^{-}$ in survey stations showed consistent patterns with lower concentrations in the top 50 m, which increased until the depth of 1,000 m and slowly decreased beyond 1,000 m. Concentrations of DSi were low in the top 50 m except S10-01 and increased until 2,000 m along with the depth. The maximum DSi concentration was 144.441 μmol/L at a depth of 2,890 m in site E87-31. ${\text{NO}}_{2}^{-}$ and ${\text{NH}}_{4}^{+}$ concentrations ranged from 0 to 0.638 μmol/L and from 0.006 to 0.634 μmol/L, respectively (Supplementary Table S1).

### Vertical abundance of *Vibrio* spp. of two different lifestyles

**Figure 2:**
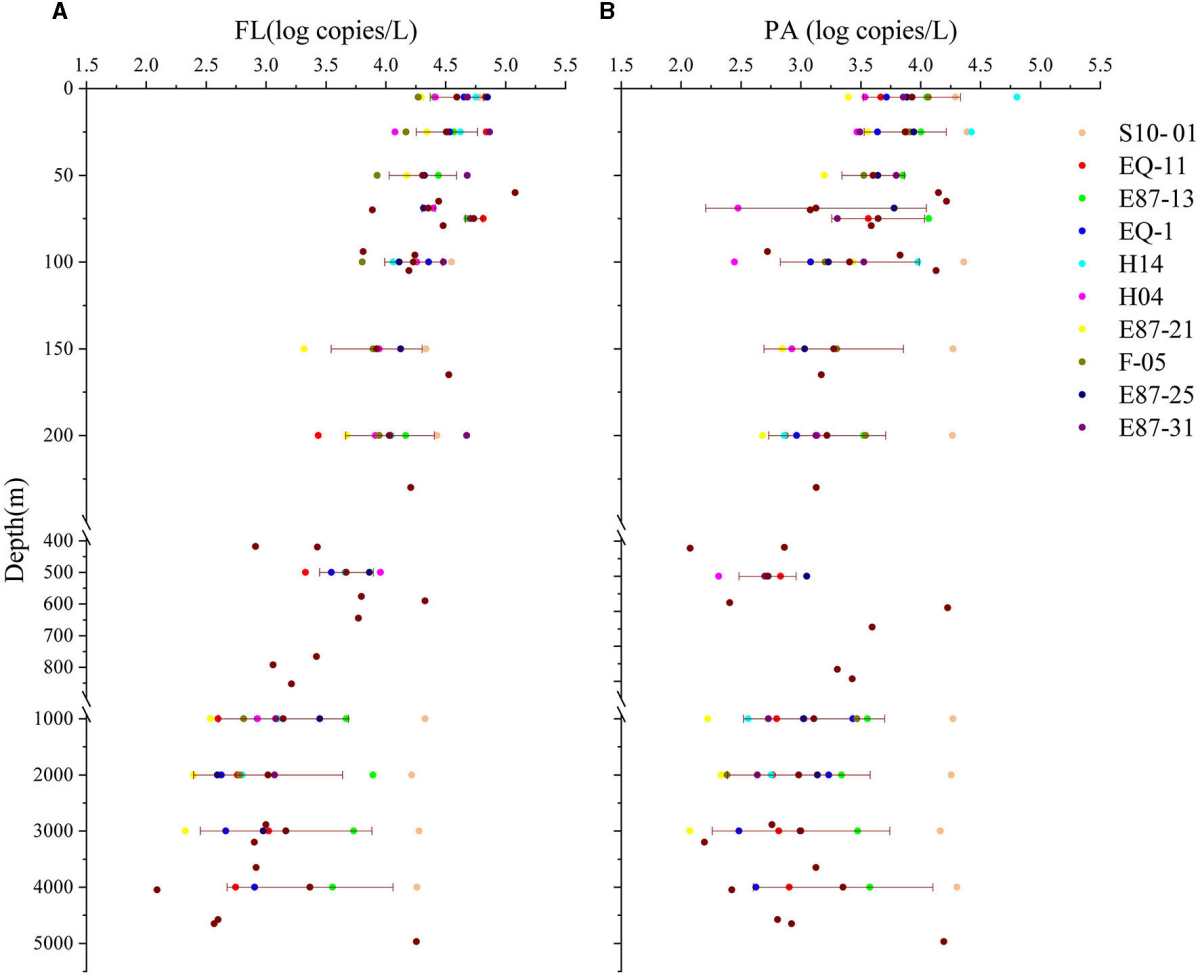
The *Vibrio* abundance of two lifestyles. The *Vibrio* abundance (log copies/liter) in all sampling water depths is shown above. **(A, B)** Represent samples in the free-living group (FL) and particle-associated group (PA). Transverse lines represent means ± SD for the number of samples at depth ≥5 m.

Quantitative PCR was used to detect the abundances of FL and PA *Vibrio* on different pore-size membranes. Overall, the abundance of *Vibrio* in the FL group was significantly much higher than that in the PA group (*p* < 0.01), and both were negatively correlated to depth (*p* < 0.001). Further analysis showed that the abundance of FL *Vibrio* was significantly much higher than PA *Vibrio* in waters above 2,000 m. The *Vibrio* abundance (log value) obviously reduced with the increasing depth of water until 2,000 m, regardless of FL and PA fractions, and this trend was even stronger in the FL group. There was no significant difference between the *Vibrio* abundances of both lifestyles in more than 2,000 m of water. The means of *Vibrio* abundance slightly raised from 2,000 m to the bottom (Figure 2). For FL *Vibrio* spp., the abundance ranged from 1.2 × 10^2^ (depth 4,050 m in site E87-21) to 1.2 × 10^5^ (depth 60 m in site H14) copies/L. As for the *Vibrio* associated with marine particles, the abundance ranged from 1.2 × 10^2^ (depth 3000 m in site E87-21) to 6.4 × 10^4^ (depth 5 m in site H14) copies/L (Supplementary Figure S1).

### Diversity analysis of the *Vibrio* community

**Figure 3:**
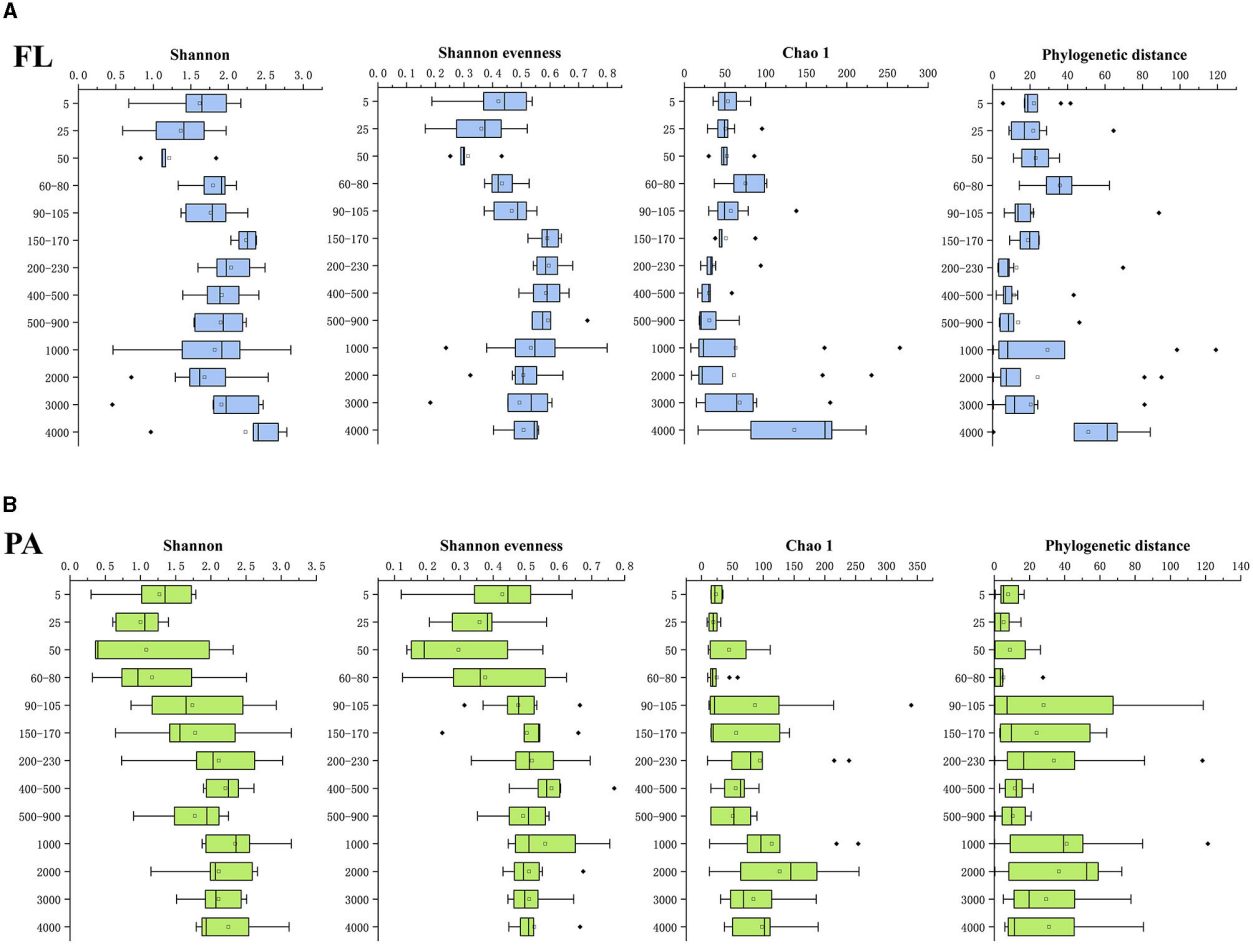
The diversity indices of FL **(A)** and PA **(B)** *Vibrio* community among the samples by water depth. FL and PA represent the free-living group and particle-associated group. The horizontal axis shows diversity indices, and the vertical axis shows the depth (m). Bars delineate the medians, the quadrate defines the mean, the hinges represent the lower and upper quartiles, the whiskers extend to the most extreme values (no more than 1.5 times the interquartile range from the box), and outliers are plotted as black diamond, if present.

The diversity among the *Vibrio* community was estimated via Illumina sequencing of the V2–V4 hypervariable regions of the 16S rRNA gene. The Illumina sequencing generated 8511917 overlapped reads ranging from 14,984 to 1,42,675 in all samples. A total of 13,971 sequences were reserved in each sample after quality control and rarefication. From the total number of sequences, we obtained 1,750 OTUs at a 97% sequence similarity threshold, which included 1,325 OTUs in FL and 841 OTUs in PA samples. Good's coverage values of all samples were > 99%, indicating that the real situation of the majority of the *Vibrio* community in the samples could be reflected by the current sequencing results. The phylogenetic distance, Chao 1 (a measure of richness), Shannon (including both evenness and diversity) index, and Shannon evenness were calculated to estimate α-diversity (Figure 3). There are similar Shannon index and Shannon evenness trends in the FL and PA groups, showing an increase with depth (Figure 3). In detail, the Shannon index ranged from 0.453 (depth 3,000 m in site E87-13) to 2.831 (depth 1,000 m in site E87-21) in the FL group and 0.299 (depth 5 m in site H4) to 3.143 (depth 150 m in site E87-21) in the PA group. The Chao 1 ranged from 8 (depth 1,000 m in site H4) to 265 (depth 1,000 m in site EQ-11) in the FL group and 8 (depth 4,970 m in site S10-01) to 340 (depth 100 m in site H4) in the PA group. As for phylogenetic distance, it registered a maximum of 119.139 (depth of 1,000 m in site EQ-11) in the FL group and 121.352 (depth of 1,000 m in site E87-25) in the PA group, and a minimum of 0.401 (depth of 1,000 m in site EQ-1) in the FL group and 0.077 (depth of 4970 m in site S10-01) in the PA group. The Shannon evenness ranged from 0.165 (depth of 25 m in site H4) to 0.799 (depth of 1,000 m in site EQ-1) in the FL group and 0.120 (depth of 5 m in site H4) to 0.768 (depth of 500 m in site H14) in the PA group.

**Figure 4:**
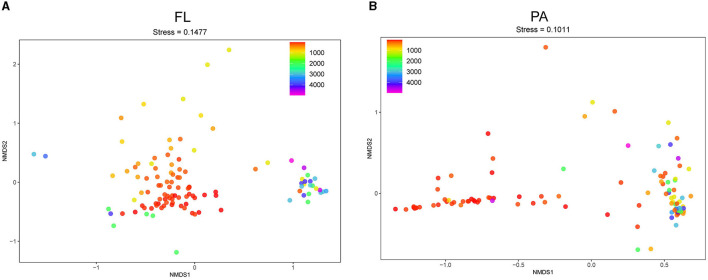
NMDS analysis of *Vibrio* spp. at the OTU level. NMDS plot of FL **(A)** and PA **(B)** group. FL, free-living group; PA, particle-associated group. The dot color gradient suggests sampling depth.

Analysis of β-diversity (non-metric multidimensional scaling, NMDS) based on the OTU level was performed to compare the *Vibrio* community composition between different samples. The stress values of NMDS analyses < 0.2 indicated that the clustering plots had considerable significance in illustrating the diversity pattern in *Vibrio* spp. across all samples (Figures 4A, B). The results demonstrated that the dots (representing samples) were distributed along with the depth.

### The compositions and vertical variations in the *Vibrio* community

**Figure 5:**
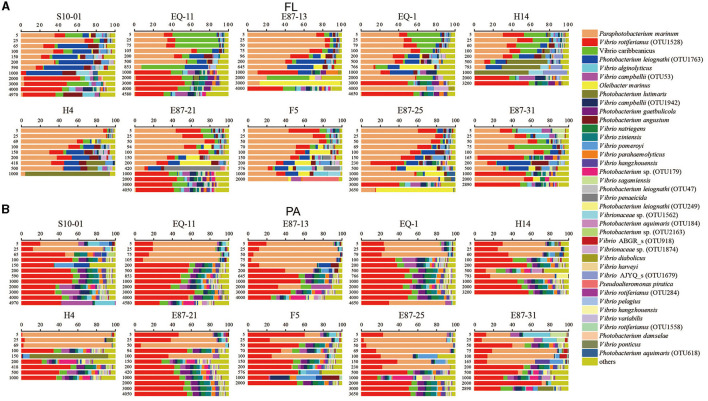
The community compositions of *Vibrio* at the species level. The figure displays the distribution of *Vibrio* species (only the 29 most abundant species were included) along with the depth at the different sites. **(A, B)** Represent samples in the free-living group (FL) and particle-associated group (PA). The horizontal axis indicates the relative abundance of the 29 most abundant species; the vertical axis indicates the depth of each station.

According to the taxonomic assignment of the *Vibrio* community conducted by comparing the representative sequences of each OTU against the SILVA database and EzBioCloud database, all the sequences primarily belonged to the *Vibrionaceae* family (more than 95%). The 29 most abundant species among the FL and PA groups of *Vibrio* communities were picked and displayed in bar graphs (Figures 5A, B). *Vibrio parahaemolyticus*, as an important pathogen, was detected in 102 of 111 samples in all sites, whereas *V. cholerae* and *V. vulnificus* were not detected in any samples.

In the FL group from the 0.22-μm PC membranes (Figure 5A), the dominant species was *Paraphotobacterium marinum* (33.84% of all sequences), followed by *V. rotiferianus* OTU1528 (16.65%), *V. caribbeanicus* (8.43%), and *Photobacterium leiognathi* OTU1763 (7.57%), each of which accounted for more than 5% and together accounted for 32.66% of all sequences. The residual 25 relatively abundant species, from *Oleibacter marinus* to *P. damselae*, comprised 26.89% of all sequences in total (0.21%−4.10%). The relative abundance of different *Vibrio* spp. changed with depth in the FL group. The relative abundance of *P. marinum* exhibited an evident increase from 5 to 25 m and a subsequent decrease depending on the site. The relative abundance of *P. marinum* was negatively correlated to depth (*r* = −0.672, *p* < 0.01). However, some sites in deeper waters had a high abundance of *P. marinum* (EQ-1, F5, E87-25, E87-31). On the other hand, *V. caribbeanicus* showed a relative decrease in abundance, moving from the superficial zone to deeper zones. However, *V. rotiferianus* OTU1528 registered a higher relative abundance in more than 2,000 m of water.

For the PA group from the 3 μm PC membranes (Figure 5B), the most abundant species was *V. rotiferianus* OTU1528, comprising 35.28% of all sequences, followed by *P. marinum*, representing 24.88% of the total sequences from PA *Vibrio*. The remnant of 27 relatively abundant species, from *V. caribbeanicus* to *V. rotiferianus* OTU1558, of which each proportion was <5%, comprised 34.43% of all sequences in total (0.25%−3.97%). PA communities were dominated by *V. rotiferianus* OTU1528 and *P. marinum*. The relative abundance of *P. marinum* was negatively correlated to depth (*r* = −0.710, *p* < 0.01), while the relative abundance of *V. rotiferianus* OTU1528 was positively correlated to depth (*r* = 0.515, *p* < 0.01). Furthermore, the dominant species of the PA group alternated from *P. marinum* in upper water to *V. rotiferianus* OTU1528 in deeper waters, depending on the site.

Two co-occurrence networks were built based on Spearman's rank correlations across all samples of two lifestyles from different depths (Supplementary Figure S2). All possible Spearman's correlation coefficients (*r* > |0.6|) between OTUs with more than 20 sequences were calculated to construct the networks. For FL (Supplementary Figure S2A), the resulting network consisted of 395 nodes linked by 1,397 edges (average degree or node connectivity 7.073; Supplementary Table S2). For PA (Supplementary Figure S2B), there were 235 nodes linked by 895 edges in the co-occurrence network (average degree or node connectivity 7.617). The majority of edges (FL: 99.86%, PA: 97.88%) in the networks were positive, indicating a predominantly cooperative relationship across the microbial community, although the co-occurrence network was limited in reflecting true interactions (Liu et al., 2019a).

### The effect of physicochemical parameters on the *Vibrio* community

**Figure 6:**
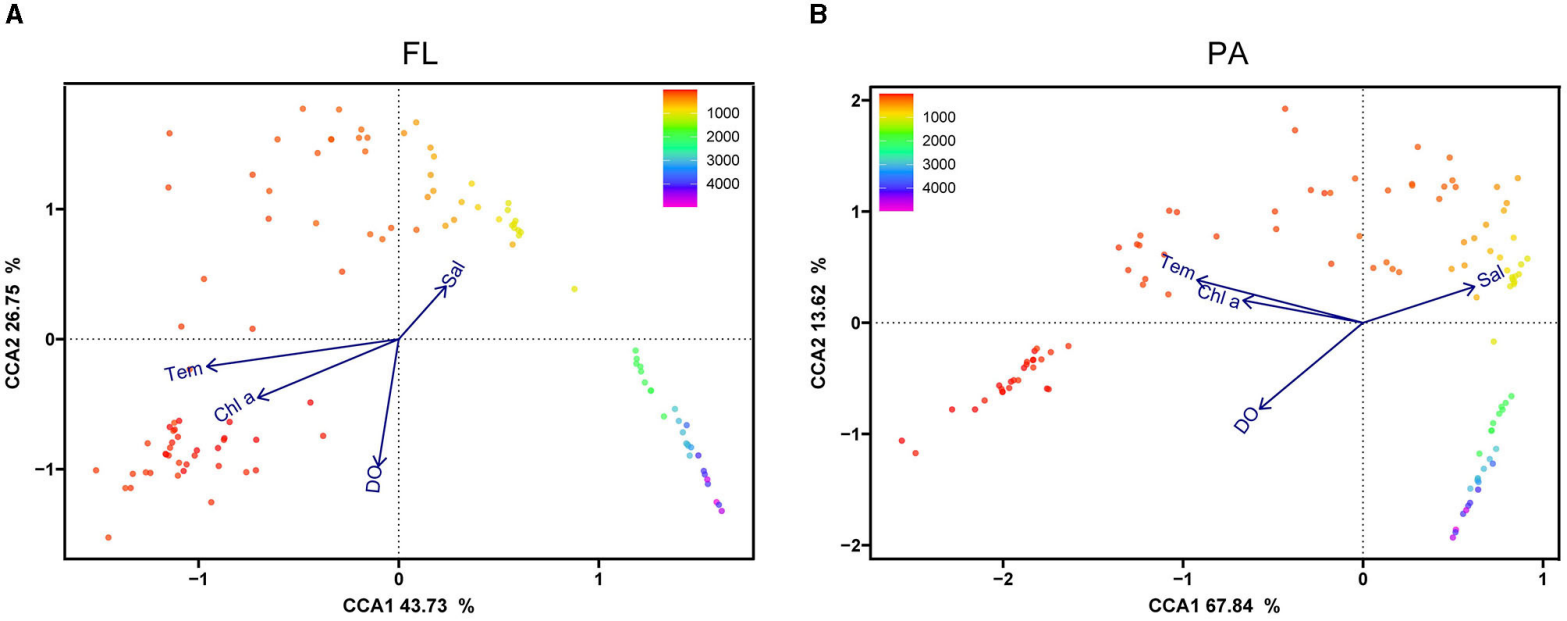
CCA analysis of *Vibrio* spp. at the OTU level. CCA illustrates the relationship between FL **(A)** and PA **(B)** *Vibrio* community at the OTU level, and the top environmental variables are shown above. FL, free-living group; PA, particle-associated group. The dot color gradient indicates sampling depth.

The canonical correspondence analysis (CCA) demonstrated the relationship between environmental factors and *Vibrio* community components at the OTU level, as indicated by the dot color gradient with sampling depth. For the CCA of samples from the 0.22-μm pore PC membranes (FL), the first axis explained 43.73% of the total variance, and the second axis explained 26.75% (Figure 6A). The appraisal of all factors performed by the Monte Carlo permutation tests (MCPT) showed that temperature, salinity, depth, and DO significantly influenced the distribution of the FL group. In the CCA diagram for the PA group, CCA1 explained 67.84% of the total variation, while CCA2 explained 13.62% (Figure 6B). The results showed that the water samples clustered more strongly by depth than by sites. Furthermore, the temperature was the most important factor that influenced the *Vibrio* community structure in the upper water, which was distinguished from other groups, and the low concentrations of DO distinguished the middle layers of water from others.

The correlations between abundance (*Vibrio* spp. in two different lifestyles) and environmental parameters were calculated by Spearman's rank correlation coefficients (Table 2). The FL *Vibrio* abundances were positively correlated with temperature (*r* = 0.765, *p* < 0.001), DO (*r* = 0.377, *p* < 0.001), Chl *a* (*r* = 0.739, *p* < 0.001), and ${\text{NO}}_{2}^{-}$ (*r* = 0.644, *p* < 0.001) but negatively correlated with salinity (*r* = −0.492, *p* < 0.001), depth (*r* = −0.788, *p* < 0.001), ${\text{NO}}_{3}^{-}$ (*r* = −0.677, *p* < 0.001), DIP (*r* = −0.752, *p* < 0.001), and DSi (*r* = −0.773, *p* < 0.001). Similarly, PA *Vibrio* spp. showed positive correlations with temperature (*r* = 0.452, *p* < 0.001), DO (*r* = 0.436, *p* < 0.001), Chl *a* (*r* = 0.417, *p* < 0.001), and ${\text{NO}}_{2}^{-}$ (*r* = 0.397, *p* < 0.001) and negatively correlated with salinity (*r* = −0.483, *p* < 0.001), water depth (*r* = −0.498, *p* < 0.001), ${\text{NO}}_{3}^{-}$ (*r* = −0.424, *p* < 0.001), DIP (*r* = −0.496, *p* < 0.001), and DSi (*r* = −0.492, *p* < 0.001).

**Table 2:** Spearman rank correlations of *Vibrio* abundance with physicochemical variables between two lifestyles.

**Variables**	**FL**			**PA**		
	***r***	***p*-value**	***n***	***r***	***p*-value**	***n***
Temperature	**0.765**	**0.000**	**111**	**0.452**	**0.000**	**111**
Salinity	**−0.492**	**0.000**	**111**	**−0.483**	**0.000**	**111**
DO	**0.377**	**0.000**	**111**	**0.436**	**0.000**	**111**
Depth	**−0.788**	**0.000**	**111**	**−0.498**	**0.000**	**111**
Chl *a*	**0.739**	**0.000**	**111**	**0.417**	**0.000**	**111**
${\text{NO}}_{3}^{-}$	**−0.677**	**0.000**	**97**	**−0.424**	**0.000**	**97**
${\text{NO}}_{2}^{-}$	**0.644**	**0.000**	**108**	**0.397**	**0.000**	**108**
${\text{NH}}_{4}^{+}$	0.081	0.438	94	0.11	0.293	94
DIP	**−0.752**	**0.000**	**107**	**−0.496**	**0.000**	**107**
DSi	**−0.763**	**0.000**	**107**	**−0.492**	**0.000**	**107**

Significant correlations (p < 0.05) are indicated in boldface.

FL, free-living group; PA, particle-associated group; r, correlation; p, significance values; n, number of samples; DO, dissolved oxygen; Chl a, chlorophyll a; DIP, dissolved inorganic phosphorus; DSi, dissolved silicon.

The Spearman correlations between the relative abundance of the top 29 most abundant species and the environmental parameters among the two lifestyles were calculated (Table 3). *Paraphotobacterium marinum*, the most abundant species, had a significant strong positive correlation (*r* = 0.693, *p* < 0.01) with temperature and positive correlation (*r* = 0.668, *p* < 0.01) with Chl *a* in the FL group. *Vibrio rotiferianus* OTU1528 negatively correlated with temperature (*r* = −0.513, *p* < 0.01) and Chl *a* (*r* = −0.535, *p* < 0.01) in the PA group. Further, TITAN was conducted to identify the response of individual taxa to the environmental gradient (Supplementary Figure S3). For temperature, a filtered sum (negative, *z*–) peak was at 6.67°C and a filtered sum (positive, *z*+) peak was at 24.53°C, and the threshold range was 17.86°C in the FL group (Supplementary Figure S3A). For DO, the filtered sum (*z*–) peak was at 2.75 mg/L, and a filtered sum (*z*+) peak was at 5.13 mg/L, and the threshold range was 2.38 mg/L in FL (Supplementary Figure S3C). Only a few OTUs showed positive responses (*z*+) to temperature and DO in PA communities, so threshold ranges of temperature and DO for PA *Vibrio* could not be validly speculated (Supplementary Figure S3).

**Table 3:** Spearman's rank correlation coefficients between the percentage composition of taxa and environmental factors.

	**Physicochemical parameters**									
	**Depth**	**Temperature**	**Salinity**	**DO**	**Chl** ***a***	**NH4** ^+^	**NO3** ^−^	** ${\text{NO}}_{2}^{-}$ **	**DIP**	**DSi**
**Free-living group**										
*Paraphotobacterium marinum* (OTU1586)	**−0.672**	**0.693**	−0.238	**0.332**	**0.668**		**−0.637**	**0.494**	**−0.661**	**−0.695**
*Vibrio rotiferianus* (OTU1528)										
*Vibrio caribbeanicus* (OTU2042)			**−0.310**	**0.400**	0.207		**−0.279**	0.237	**−0.287**	−0.213
*Photobacterium leiognathi* (OTU1763)	**−0.250**	0.227	**0.395**	**−0.528**	0.206	**0.299**				**−0.252**
*Oleibacter marinus* (OTU619)				−0.196						
*Photobacterium angustum* (OTU2053)	**−0.384**	**0.366**	0.234	**−0.356**	**0.376**	**0.275**	**−0.318**	0.233	**−0.314**	**−0.401**
*Photobacterium lutimaris* (OTU926)	0.218	−0.234		−0.238	−0.190			**−0.281**	**0.273**	0.215
*Vibrio alginolyticus* (OTU833)			**−0.318**	**0.386**			−0.227		**−0.260**	
*Vibrio campbellii* (OTU53)				0.233						
*Vibrio pomeroyi* (OTU674)	**−0.404**	**0.366**			**0.437**		**−0.426**	**0.269**	**−0.410**	**−0.388**
*Vibrio campbellii* (OTU1942)										
*Vibrio natriegens* (OTU767)			−0.197							
*Photobacterium leiognathi* (OTU47)	−0.195				0.215	**0.271**	**−0.293**	0.197	−0.215	−0.196
*Vibrio ziniensis* (OTU1810)			−0.195	**0.245**						
*Vibrio sagamiensis* (OTU214)	**−0.260**	**0.254**	**−0.347**	**0.344**	**0.323**		−0.259	**0.281**	**−0.287**	−0.226
*Vibrio penaeicida* (OTU438)	0.202	−0.191							0.193	**0.307**
*Vibrionaceae* sp. OTU1562			**0.281**	**−0.380**		0.231				
*Photobacterium gaetbulicola* (OTU2)	**0.475**	**−0.483**		−0.232	**−0.428**		**0.387**	**−0.388**	**0.477**	**0.470**
*Vibrio parahaemolyticus* (OTU542)	0.203	−0.188								
*Vibrionaceae* sp. OTU1874			0.234	**−0.344**				−0.202		
*Pseudoalteromonas piratica* (OTU391)					0.251			0.213		
*Vibrio* AJYQ_s (ZF-129) (OTU1679)						0.223				
*Vibrio* ABGR_s (AND4) (OTU918)	**−0.398**	**0.374**	**−0.248**		**0.353**		**−0.328**	**0.314**	**−0.355**	**−0.344**
*Vibrio hangzhouensis* (OTU932)	**0.383**	**−0.386**			**−0.323**		0.220	−0.237	**0.289**	**0.399**
*Vibrio pelagius* (OTU1124)	**0.361**	**−0.373**			**−0.289**			−0.246	**0.261**	**0.350**
*Photobacterium aquimaris* (OTU618)			**0.246**	**−0.337**	−0.239			**−0.282**	0.220	
*Photobacterium* sp. OTU179	**0.518**	**−0.519**			**−0.436**		**0.306**	**−0.271**	**0.380**	**0.532**
*Vibrio diabolicus* (OTU759)	**0.294**	**−0.312**			−0.226				0.221	**0.30**
**Particle-associated group**										
*Vibrio rotiferianus* (OTU1528)	**0.515**	**−0.513**	**0.308**	**−0.280**	**−0.535**		**0.370**	**−0.421**	**0.468**	**0.499**
*Paraphotobacterium marinum* (OTU1586)	**−0.710**	**0.716**	**−0.443**	**0.452**	**0.724**		**−0.639**	**0.643**	**−0.688**	**−0.712**
*Vibrio caribbeanicus* (OTU2042)	**0.518**	**−0.520**	**0.369**	**−0.275**	**−0.503**		**0.390**	**−0.450**	**0.478**	**0.510**
*Vibrio campbellii* (OTU53)								−0.202		
*Photobacterium gaetbulicola* (OTU2)	**0.531**	**−0.532**	**0.386**	**−0.361**	**−0.494**		**0.433**	**−0.469**	**0.521**	**0.520**
*Vibrio campbellii* (OTU1942)	**0.278**	**−0.285**	0.242		**−0.309**		0.229	**−0.344**	**0.271**	**0.255**
*Vibrio natriegens* (OTU767)	**0.478**	**−0.489**	**0.286**	**−0.272**	**−0.484**		**0.387**	**−0.398**	**0.455**	**0.483**
*Vibrio ziniensis* (OTU1810)	**0.439**	**−0.453**	**0.259**	**−0.214**	**−0.447**		**0.365**	**−0.335**	**0.430**	**0.459**
*Vibrio alginolyticus* (OTU833)				0.193						
*Vibrio parahaemolyticus* (OTU542)	**0.516**	**−0.533**	**0.367**	**−0.337**	**−0.508**		**0.468**	**−0.420**	**0.536**	**0.547**
*Photobacterium* sp OTU179	**0.482**	**−0.496**	**0.410**	**−0.404**	**−0.422**		**0.445**	**−0.425**	**0.521**	**0.493**
*Vibrio hangzhouensis* (OTU932)	**0.468**	**−0.476**	**0.389**	**−0.400**	**−0.397**		**0.414**	**−0.361**	**0.488**	**0.478**
*Vibrio pomeroyi* (OTU674)		−0.216	**0.292**	**−0.413**	−0.198			−0.210	0.248	0.191
*Photobacterium lutimaris* (OTU926)	**0.366**	**−0.383**	**0.286**	**−0.295**	**−0.345**		0.219	**−0.325**	**0.346**	**0.317**
*Vibrio sagamiensis* (OTU214)										
*Photobacterium leiognathi* (OTU1763)			0.198							
*Photobacterium leiognathi* (OTU249)	**0.527**	**−0.526**	**0.424**	**−0.380**	**−0.495**		**0.458**	**−0.434**	**0.562**	**0.518**
*Photobacterium aquimaris* (OTU184)	**0.485**	**−0.488**	**0.436**	**−0.402**	**−0.453**		**0.447**	**−0.413**	**0.533**	**0.474**
*Photobacterium leiognathi* (OTU47)	**0.408**	**−0.417**	**0.412**	**−0.321**	**−0.330**		**0.337**	**−0.380**	**0.403**	**0.401**
*Photobacterium* sp OTU2163	**0.496**	**−0.510**	**0.325**	**−0.322**	**−0.483**		**0.416**	**−0.411**	**0.520**	**0.506**
*Vibrio harveyi* (OTU1559)	**0.557**	**−0.562**	**0.383**	**−0.339**	**−0.535**		**0.453**	**−0.471**	**0.568**	**0.546**
*Photobacterium angustum* (OTU2053)	−0.237	0.206			**0.290**				−0.222	**−0.258**
*Vibrio diabolicus* (OTU759)	**0.521**	**−0.517**	**0.423**	**−0.364**	**−0.498**		**0.390**	**−0.426**	**0.515**	**0.492**
*Vibrio hangzhouensis* (OTU1650)	**0.472**	**−0.461**	**0.357**	**−0.344**	**−0.446**		**0.409**	**−0.378**	**0.506**	**0.481**
*Vibrio variabilis* (OTU262)	**0.552**	**−0.552**	**0.384**	**−0.353**	**−0.545**		**0.451**	**−0.468**	**0.553**	**0.550**
*Vibrio rotiferianus* (OTU284)	0.234	−0.228	0.196		**−0.294**			**−0.294**	0.196	0.201
*Vibrio* ABGR_s (AND4) (OTU918)	−0.221	0.196	−0.202							
*Vibrio ponticus* (OTU359)	**0.540**	**−0.542**	**0.376**	**−0.289**	**−0.499**		**0.395**	**−0.379**	**0.501**	**0.532**

Only significant correlations (p < 0.05) are shown in the table. –, negative correlation; Bold, p < 0.01; regular, p < 0.05.

FL, free-living group; PA, particle-associated group; DO, dissolved oxygen; Chl a, chlorophyll a; DIP, dissolved inorganic phosphorus; DSi, dissolved silicon.

## Discussion

Distribution patterns and abundance of *Vibrio* species in different marine environments have been studied previously (Zhang et al., 2018). However, most of these works have focused on the estuary or offshore zone which is near the coastline, and those studies have been limited to the surface layer (Siboni et al., 2016; Zhang et al., 2018; Liang et al., 2019; Wang et al., 2020). In this study, the ecological distribution patterns of *Vibrio* communities were characterized based on the varied *Vibrio* lifestyles along a vertical environment variation in the ETIO. The results revealed that community compositions of *Vibrio* populations in free-living and particle-associated lifestyles exhibit a significant stratified distribution pattern based on dynamic environment factors; the preferential lifestyles shifted from free-living to mixed as depth increased. The results of this study help supplement knowledge on *Vibrio* distribution patterns affected by environmental factors in the oceans and offer directions to further explore the ecological roles of *Vibrio* in the open seas.

### Vibrios may shift from free-living to mixed lifestyles from the surface to bottom seawater in the ETIO

It has been reported that PA strategy is the major lifestyle of *Vibrio* spp. in most cases, and FL lifestyle may help vibrios find appropriate trophic niches through chemotactic motility (Brennan et al., 2013; Wang et al., 2022). In this study, the FL vibrios were predominant position in water samples above 2,000 m (*p* < 0.001). In contrast, in waters deeper than 2,000 m, there was no significant difference between the abundance of FL and PA vibrio. These results were in line with the results of other studies in the shallow sea, such as the Baltic and Skagerrak Seas, the Mediterranean, and the northern Chinese marginal seas (Eiler et al., 2006; Bellés-Garulera et al., 2016; Liang et al., 2019). Further, the nutrient limitations in the ETIO may be responsible for the *Vibrio* community with FL as its temporary dominant lifestyle in waters above 2,000 m (Ning et al., 2004; Huang et al., 2019). In the present study, FL *Vibrio* abundance decreased more rapidly than PA during an increase in water depth due to changes in environmental factors (Figure 2). The reason might be that FL *Vibrio* was more susceptible to changes in environmental factors (especially temperature) than the PA group (Table 2). Additionally, the means of FL *Vibrio* abundance slightly raised from 2,000 m to the bottom, possibly due to the resuspension of organic matter from the surface sediment (Vezzulli et al., 2009; Qian et al., 2018).

Various environmental determinants may play potential roles in determining whether *Vibrio* remains free-living or particle-associated, such as temperature, pH, salinity, and ion concentration (Hood and Winter, 1997; Hsieh et al., 2007; Takemura et al., 2014). The decrease in temperature with depth may be the main explanation, which is usually a vital factor for the changed abundance of *Vibrio* (Oberbeckmann et al., 2012; Froelich et al., 2013). Both FL and PA vibrios showed positive correlations with temperature, and the correlation coefficient of FL vibrios with temperature was higher than that in the PA group (Table 2). Consequently, this also means that rising ocean surface temperatures could increase in *Vibrio* abundance in seawater that threatens the health of human and aquaculture animals (Siboni et al., 2016). DO is an important hydrological parameter that affects the abundance of the *Vibrio* population by switching from respiration to fermentation mode (Li et al., 2020). Negative correlations between the abundance of *Vibrio* and DO have been found on the coast of Georgia (USA), North Carolina estuaries (USA), and Sansha Yongle Blue Hole (China) (Blackwell and Oliver, 2008; Turner et al., 2009; Li et al., 2020). There was a positive correlation between DO and *Vibrio* abundance in the ETIO, and the reason might be that the concentration range of DO was broad (Supplementary Table S1). Other environmental factors (e.g., salinity) may also have some influence but reveal no clear trends. Further, large particulate matter settling into the deep sea may provide potential nutrient sources for vibrios to attach to Verdugo et al. (2004). Deposition of particulate matter from upper water can explain the higher abundance ratio of PA to FL vibrios in waters more than 2,000 m in depth (Mestre et al., 2017; Kopprio et al., 2020). Taken together, as an opportunistic bacteria, *Vibrio* chooses appropriate growth strategies to maintain the population in response to changes in the environment. However, factors determining whether *Vibrio* remains free-living or particle-associated are still unclear, and further studies (e.g., genetic determinants) should be conducted in the future.

As expected, the abundance of *Vibrio* averaged 1.8 × 10^4^ copies/liter in all FL samples and 5.3 × 10^3^ copies/liter in all PA samples, which were lower than that in the marginal seas (Liang et al., 2019; Wang et al., 2020; Xu et al., 2020; Williams et al., 2022). The possible reason for this disparity could be the varying amounts of terrigenous nutrients that can be introduced into estuaries, nearshore waters, and marginal sea, whereas the sampling area in ETIO is a typical oligotrophic environment that does not have inputs of terrestrial resources (Liang et al., 2019; Wu et al., 2019; Wang et al., 2020; Xu et al., 2020; Williams et al., 2022). The different copy numbers of the 16S rRNA gene among species (from 2 to 21 operons in vibrios) may also contribute to the low abundance of vibrios measured by qPCR in the ETIO (Lin et al., 2018).

### FL vibrios may be a useful barometer for global warming and the change in DO

It has been reported that dominant species have regional distribution characteristics (Wong et al., 2019). *Vibrio campbellii, Vibrio atlanticus*, and *V. caribbeanicus* were the dominant species in the Northern Chinese Marginal Seas (Liang et al., 2019)—*V. atlanticus* and *Vibrio owensii* in the Changjiang estuary (Wang et al., 2020) and *Vibrio fluvialis* in the Maowei Sea in China (Chen et al., 2020). In the ETIO, *P. marinum* and *V. rotiferianus* OTU1528 were the most abundant species. *Paraphotobacterium marinum* and *V. rotiferianus* are highly adaptable in marine environments (Gomez-Gil et al., 2003; Huang et al., 2016). *Paraphotobacterium marinum* was considered specific to the pelagic environment and globally distributed from the surface to the deep extreme hydrothermal regions in FL or PA lifestyle (Huang et al., 2017). Meanwhile, *V. rotiferianus* was also distributed in various marine environments around the world (Wong et al., 2019; Chen et al., 2020; Wang et al., 2020; Xu et al., 2020; Zampieri et al., 2020) living freely in marine environments or as pathogenic bacteria to infect certain fish, shrimps, and reef-building corals in the Indo-Pacific (Cervino et al., 2008; Xue et al., 2017; Zhang et al., 2019). The alterations of community compositions of *Vibrio* in two lifestyles were also observed in the ETIO. Our results suggest that specific *Vibrio* species may exhibit different preferential lifestyles, allowing them to compete with other bacteria (Wang et al., 2022). The relative abundances of *V. campbellii* OTU53 and OTU1942 were high in the PA group, whereas the FL group exhibited relative abundances of *V. caribbeanicus, P. leiognathi* OTU1763, and *O. marinus*. *Vibrio campbellii* is an important pathogen that affects molluscs, finfish, and shrimp in aquaculture (Defoirdt et al., 2006). Comparing genomes revealed a hypothetical protein found in intimins that contributes to attaching and effacing lesions, which may be related to *V. campbellii*'s fitness in the PA group (Leo et al., 2015; Ke et al., 2017). In the PA group, there were only a few OTUs' relative abundance positive (*z*+) responding to temperature (4 OTUs, Supplementary Figure S3B) and DO (3 OTUs, Supplementary Figure S3D). The possible reason is that the particle-associated strategy helps vibrios weaken the effects of environmental changes to some extent (Karunasagar et al., 1996).

Given that the threshold ranges of temperature and DO for PA *Vibrio* could not be validly speculated (Supplementary Figure S3), the FL *Vibrio* community may be a better barometer for changes in temperature and DO. In the FL group, *V. caribbeanicus* was mainly distributed in waters above 200 m in certain stations (Figure 5A), which also was the most abundant *Vibrio* species in summer samples from the Bohai Sea and North Yellow Sea (Liang et al., 2019). Our TATIN analysis revealed that the relative abundance of *V. caribbeanicus* (OTU2042) positively (*z*+) responded to temperature, and the optimum temperature was 29.8, which was consistent with a previous study that considered it as a warm water species (Vezzulli et al., 2016; Liang et al., 2019). *Photobacterium leiognathi* OTU1763 was mainly present in 200–1,000 m seawater, where the concentration of DO was almost the lowest (Figure 5A, Supplementary Table S1). *Photobacterium leiognathi* can inhabit the light organs of fish as symbionts where oxygen is limited and can be discharged through the intestines into the seawater (Kaeding et al., 2007; Urbanczyk et al., 2011). Our TITAN analysis speculated that *P. leiognathi* is sensitive to oxygen concentrations and can adapt to a narrow DO range in FL samples (Supplementary Figure S3C). Though the relative abundance of *O. marinus* in the FL community varied with sites at different depths, it mainly occupied a high relative abundance in bottom seawater (Figure 5A), which may be associated with temperature, nutrients, and oil pollution in the ETIO. Liu et al. found that *Oleibacter* developed well in the nutrient-enriched bottom water of the Northern Gulf of Mexico (Liu et al., 2017). It has high abundance and plays a key role in microbial hydrocarbon degradation at seawater deeper than 10,000 m in the Mariana Trench (Liu et al., 2019b). Additionally, it is worth noting that *V. parahaemolyticus* was detected in almost all samples (Figure 5). *Vibrio parahaemolyticus* can cause severe gastroenteritis in humans through the consumption of undercooked or contaminated seafood (Zhang et al., 2018). Our sampling sites may have been one of the sources of *V. parahaemolyticus* pandemic spread (Baker-Austin et al., 2018), and further studies should be conducted in the future to determine it.

### High relative abundance of genera *Photobacterium* and *Paraphotobacterium* species may associate with coral and fish school

More than 49% of sequences in the FL group and 34% of sequences in the PA group were assigned to the genera *Photobacterium* and *Paraphotobacterium* in this study. *Paraphotobacterium marinum* was the only species in the genus *Paraphotobacterium* in 2016 (Huang et al., 2016), which was the most abundant species in FL vibrios communities and the second abundant species in PA vibrios communities in the present study. It was isolated from the surface seawater of the South China Sea for the first time (Huang et al., 2016). The wider tolerance range of temperature (15–35°C and optimum of 30°C) and salinity (1%−6% and optimum of 3%) of this species may contribute to its high proportion in the vertical water column with wide temperature range and high salinity in the ETIO (Huang et al., 2016). The symbiotic relationship between *P. marinum* and coral *Stylophora pistillata* was also discovered by comparing the 16S rRNA gene partial sequence of uncultured bacterium clone P4-A03 (KC668602) against the EzBioCloud database (Bayer et al., 2013; Huang et al., 2017). *Stylophora pistillata* is a model coral whose range spans the Indo-Pacific (Meziere et al., 2022). Meanwhile, consistent with the sedimentary *Vibrio* community in the Chinese marginal seas (Wang et al., 2019), many *Photobacterium* species occurred in the ETIO. The distribution pattern of those species may be affected by the environment and marine organisms (Moi et al., 2017). For example, *P. leiognathi* (OTU1763, OTU47) can colonize in the internal light organ of fish of the family Leiognathidae, which are widely distributed in the Eastern Indian Ocean, and form a bioluminescent symbiosis with the fish by providing light used in bioluminescence displays (McFall-Ngai and Dunlap, 1984; Dunlap and McFall-Ngai, 1987). Excess cells are released from light organs into the seawater through the gut tract of the fish with the growth of the *P. leiognathi* population (Urbanczyk et al., 2011). Additionally, *Photobacterium* and *Paraphotobacterium* species are phylogenetically close to *Vibrio* species, and the morphological and rRNA description of their species show a high degree of consistency with vibrios (Baumann et al., 1983).

Due to the limitations of the primers used in high-throughput sequencing and the high genetic similarity of *Vibrionaceae* (Sawabe et al., 2007), it has been difficult to distinguish species in the genus *Vibrio* from other genera within the *Vibrionaceae* based on 16S rRNA gene until now (Szabo et al., 2013). In addition, on account of the relatedness and rapid evolution of the *Vibrio* species, it is difficult to identify and differentiate *Vibrio* species relying on short reads of the 16S rRNA gene (Ruimy et al., 1994). The emergence of a new approach may further improve *Vibrio* research in the environment. Recently, third-generation sequencing (TGS) technologies, represented by Pacific Biosciences' single molecule real-time (SMRT) technology and Oxford Nanopore Technologies (ONT) (McCarthy, 2010; Ip et al., 2015), do not require PCR amplification and can sequence DNA fragment for long reads. It is possible to identify *Vibrio* species more accurately in the future. Except for the 16S rRNA gene, several housekeeping genes have been used as makers to identify and differentiate *Vibrio* and other bacteria with high taxonomic resolution. The heat shock protein 60 gene (*hsp60*) may be an excellent target for NGS to distinguish closely related taxa (Kwok et al., 2002; Jesser and Noble, 2018), and the use of the ferric uptake regulator (*fur*) gene and uridylate kinase (*pyrH*) gene can improve the study of *Vibrio* species (Machado and Gram, 2015; Amin et al., 2016). We have attempted to amplify these housekeeping genes from environmental samples in our lab, but the low success rate makes it difficult to apply. Experiments with the housekeeping genes to improve sequencing resolution should be attempted further in the future.

## Conclusion

The vertical distribution of *Vibrio* in the ETIO was revealed by relying on qPCR and high-throughput sequencing in this study. Overall, the abundance of *Vibrio* decreased from surface water to deep water, and the abundance of FL *Vibrio* was much higher than the PA group. *Paraphotobacterium marinum* and *V. rotiferianus* were dominant species in the water column of the ETIO. In addition, the community composition of *Vibrio* spp. varied with depth, and several important species (*P. marinum, V. caribbeanicus*, and *P. leiognathi*, for instance) were significantly correlated with the alteration of environmental factors (temperature, salinity, dissolved oxygen, nitrogen, phosphorus, and silicon). Our study paid attention to the vertical distribution of *Vibrio* spp. and its correlation with environmental factors in the ETIO and expanded the study of vibrionic ecology in open sea ecosystems. In the future, more sophisticated sequencing techniques based on housekeeping genes or full-length sequencing of 16S rRNA genes based on TGS technologies should be used to improve sequencing resolution and contribute to figuring out the roles of the *Vibrio* community in biogeochemical cycling.

## Data availability statement

The datasets presented in this study can be found in online repositories. The names of the repository/repositories and accession number(s) can be found in the article/Supplementary material.

## Author contributions

SZ: Conceptualization, Data curation, Formal analysis, Investigation, Writing—original draft, Writing—review and editing. XW: Conceptualization, Data curation, Formal analysis, Methodology, Supervision, Writing—review and editing. WZ: Data curation, Software, Visualization, Writing—original draft. YZ: Investigation, Methodology, Writing—original draft. DS: Investigation, Methodology, Writing—original draft. HC: Investigation, Writing—original draft. X-HZ: Conceptualization, Funding acquisition, Methodology, Resources, Supervision, Writing—review and editing.
