# Supplementary material for: Vertical dynamics of free-living and particle-associated vibrio communities in the eastern tropical Indian Ocean
Source: Front Microbiol. 2023 Oct 19;14:1285670. doi: 10.3389/fmicb.2023.1285670 (PMC10620696; doi:10.3389/fmicb.2023.1285670)
Supplement: Supplementary file 1 [file Data_Sheet_1.docx]

**Supplementary data for:**

**Vertical Dynamics of Free-Living and Particle-Associated *Vibrio* Communities in the Eastern Tropical Indian Ocean**

Shaodong Zhu^a†^, Xiaolei Wang^a, b†^, Wenbin Zhao^a^, Yulin Zhang^a^, Derui Song^a^, Haojin Cheng^a^, Xiao-Hua Zhang^a, b, c*^

^a^Frontiers Science Center for Deep Ocean Multispheres and Earth System, and College of Marine Life Sciences, Ocean University of China, Qingdao, China

^b^Laboratory for Marine Ecology and Environmental Science, Laoshan Laboratory, Qingdao, China

^c^Institute of Evolution and Marine Biodiversity, Ocean University of China, Qingdao, China

^†^These authors contributed equally to this work.

*Author for correspondence:

Xiao-Hua Zhang

Email: xhzhang@ouc.edu.cn

**Running title: Vertical dynamics of vibrios in Indian Ocean**


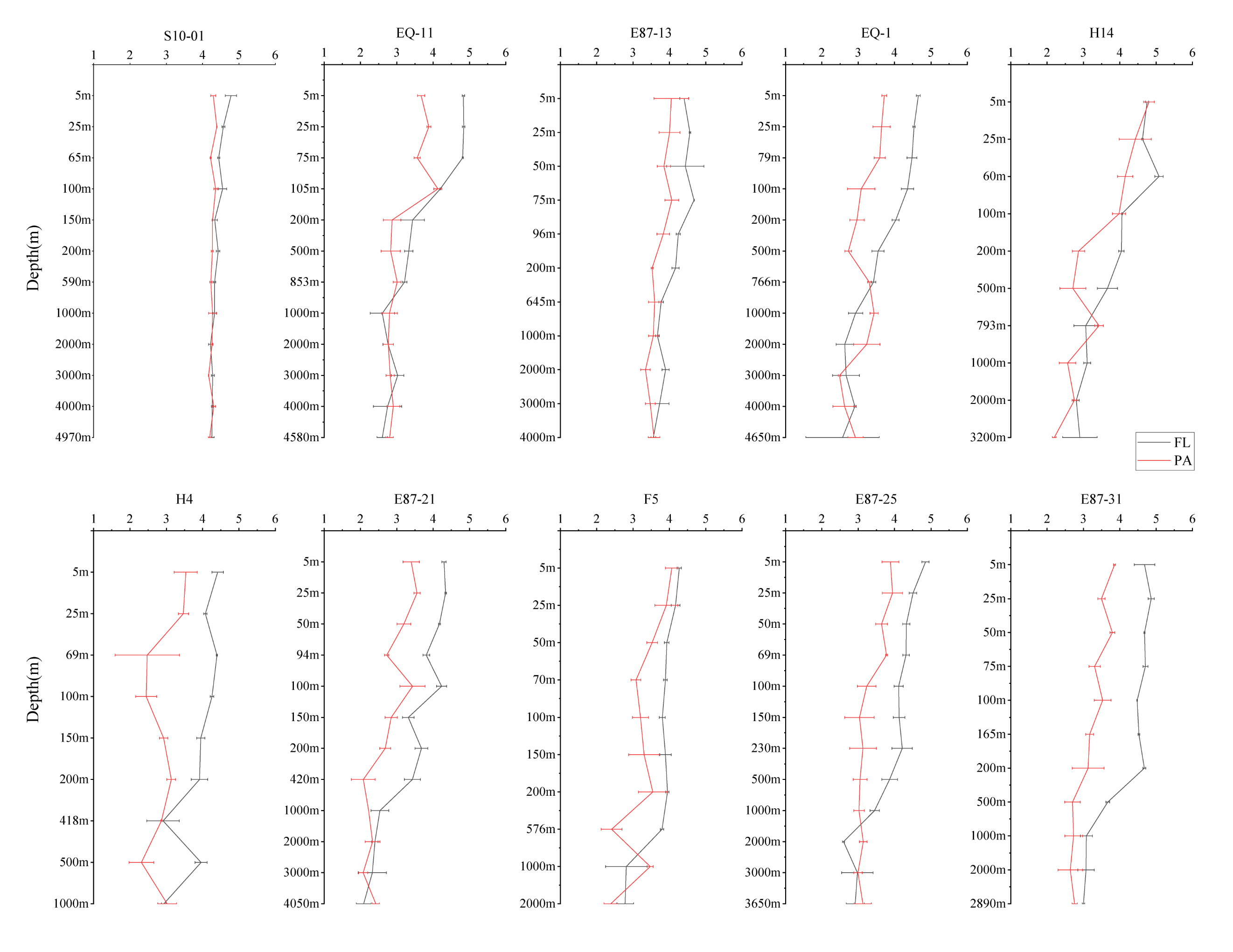


**Figure. S1 The abundance of free-living and particle-associated *Vibrio* spp. in 10 sampling sites.** The horizontal axis shows abundance (log copies/liter) and the vertical axis shows the depth (m). FL: free-living group, PA: particle-associated group.

**
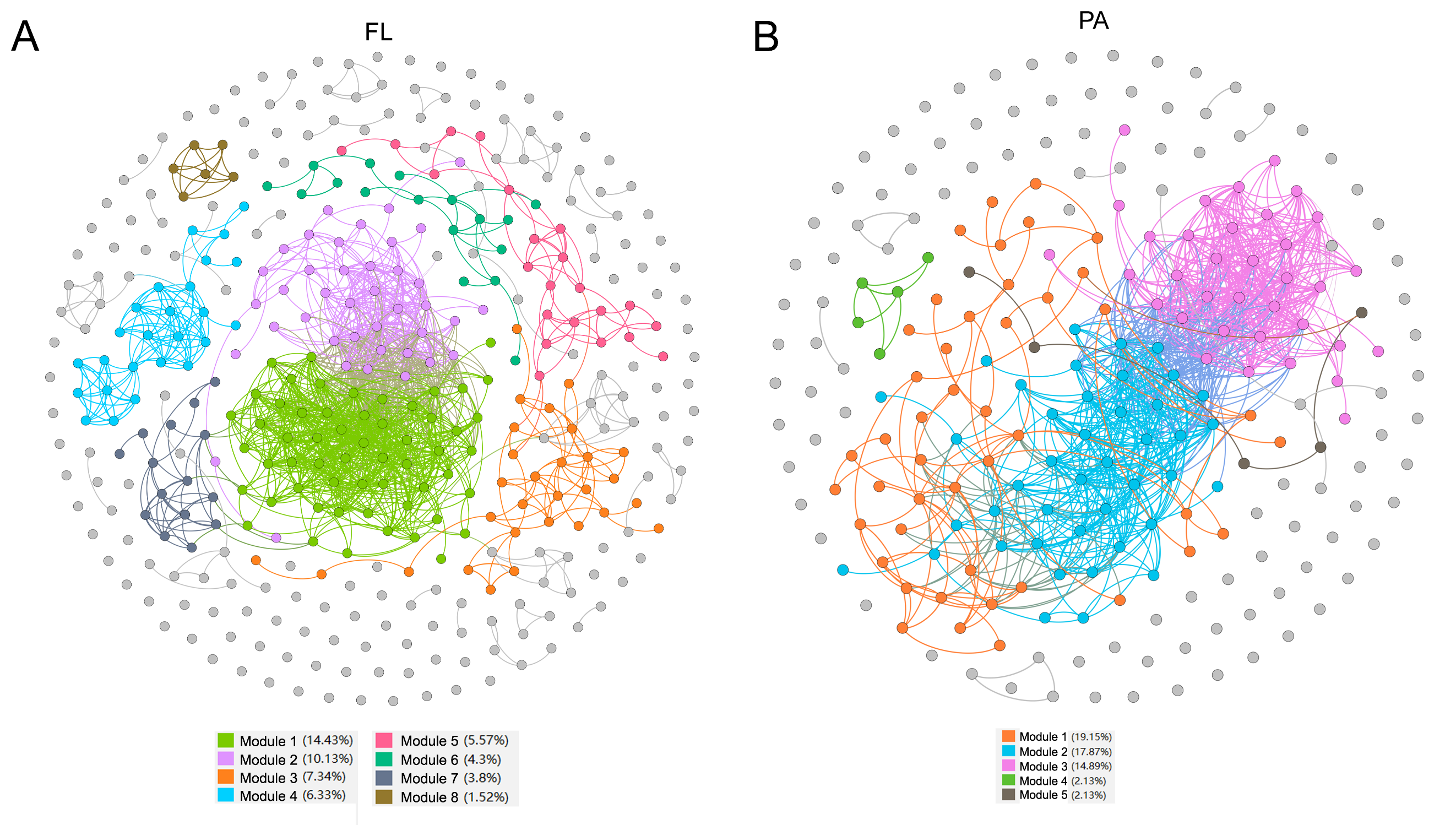
**

**Figure. S2 Co-occurrence networks of the *Vibrio* community in free-living and particle-associated samples.** A: Co-occurrence networks of FL *Vibrio* community with OTUs coloured by modularity, B: Co-occurrence networks of PA *Vibrio* community with OTUs coloured by modularity.


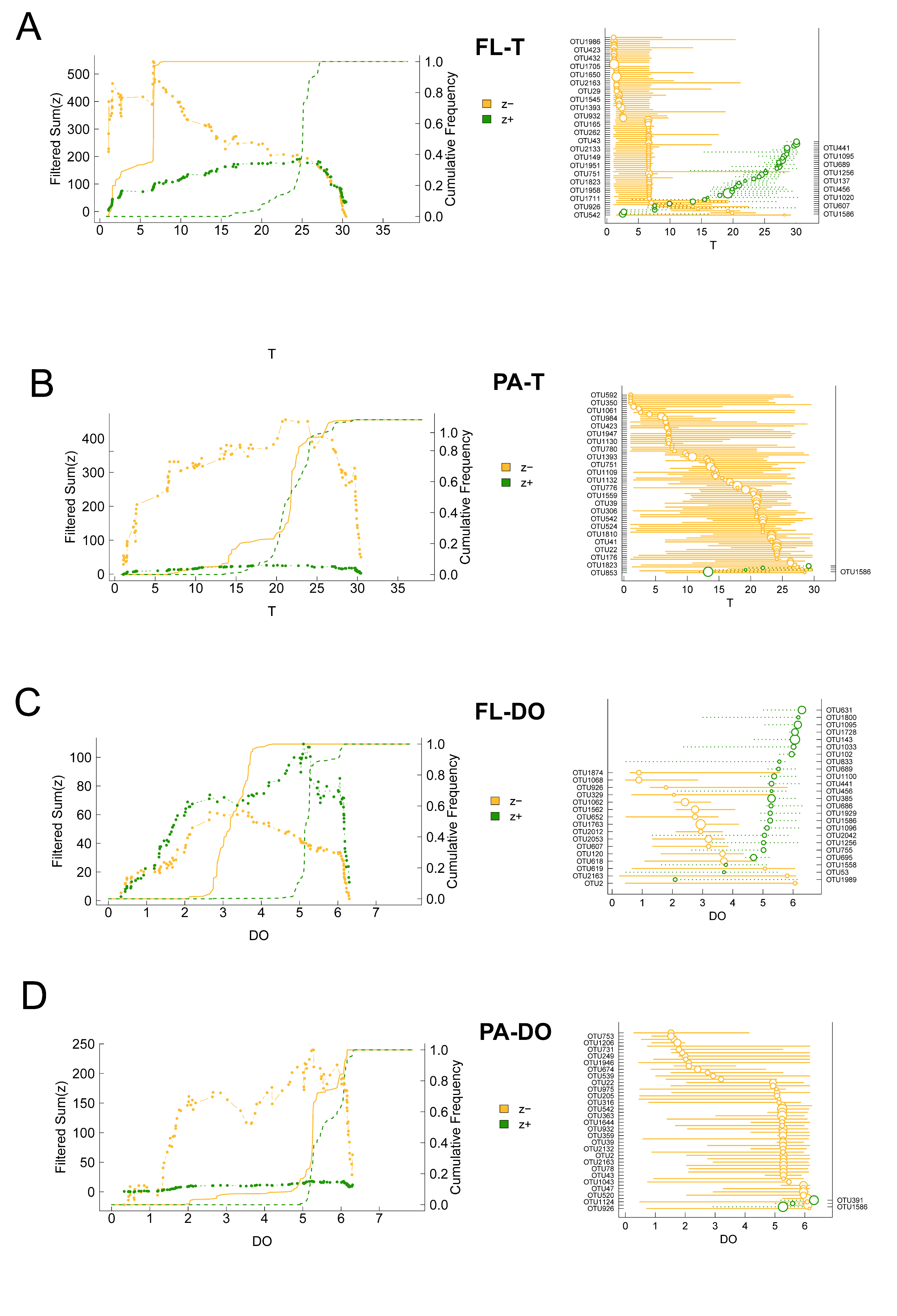


**Figure. S3 Threshold Indicator Taxa Analysis of *Vibrio* community at OTU level.**

A: FL-T, TITAN analysis of free-living community along the temperature gradient; B: PA-T, TITAN analysis of particle-associated community along the temperature gradient; C: FL-DO, TITAN analysis of free-living community along the DO gradient. D: PA-T, TITAN analysis of particle-associated community along the DO gradient. Left, Community-level sums of taxon-specific indicator scores along the temperature gradient. Right, the OTUs correlated with temperature and the predicted optimum temperature. Z+, positive response (green). Z-, negative response (yellow).

**Table S1 The environmental parameters in 10 sampling sites.**

| **Station** | **Depth**  **(m)** | **T**  **(℃)** | **Salinity (PSU)** | **DO (mg/L)** | **Chl *a* (ug/L)** | **NH_4_^+^**  **(μM)** | **NO_3_^-^ (μM)** | **NO_2_^-^ (μM)** | DIP (**μM/L)** | **DSi**  (**μM/L**) |
| --- | --- | --- | --- | --- | --- | --- | --- | --- | --- | --- |
| S10-01 | 5 | 28.056 | 34.4789 | 6.1848 | 0.143 | 0.090 | N.D. | N.D. | 0.136 | 1.883 |
|  | 25 | 27.9647 | 34.46 | 6.36 | 0.186 | 0.052 | N.D. | N.D. | 0.102 | 1.907 |
|  | 65 | 20.1092 | 34.6692 | 2.8468 | 0.434 | 0.090 | 12.746 | 0.149 | 1.184 | 21.718 |
|  | 100 | 17.0484 | 34.7258 | 2.6529 | 0.111 | 0.027 | 17.212 | 0.034 | 1.469 | 27.655 |
|  | 150 | 14.4994 | 34.7146 | 2.0705 | 0.009 | 0.065 | 19.378 | 0.038 | 1.700 | 31.948 |
|  | 200 | 12.9743 | 34.75 | 2.0218 | 0.007 | 0.363 | 21.453 | 0.009 | 1.743 | 32.859 |
|  | 590 | 7.8408 | 34.7268 | 2.0124 | 0.000 | 0.074 | 31.79 | 0.006 | 2.453 | 56.206 |
|  | 1000 | 5.3144 | 34.6642 | 2.6357 | 0.000 | 0.276 | 34.783 | 0.009 | 2.606 | 91.712 |
|  | 2000 | 2.2857 | 34.7345 | 4.6269 | 0.000 | 0.040 | 33.874 | 0.003 | 2.618 | 114.862 |
|  | 3000 | 1.5645 | 34.7255 | 5.2544 | 0.000 | 0.031 | 33.287 | 0.010 | 2.533 | 121.169 |
|  | 4000 | 1.0763 | 34.716 | 5.71 | 0.000 | 0.617 | 32.96 | 0.000 | 2.461 | 121.016 |
|  | 4970 | 0.924 | 34.7128 | 6.0105 | 0.000 | 0.042 | 31.339 | 0.014 | 2.366 | 117.509 |
| EQ-11 | 5 | 29.8528 | 34.117 | 6.1837 | 0.322 | 0.2170 | N.D. | 0.044 | 0.033 | 1.791 |
|  | 25 | 29.8991 | 34.1736 | 6.2188 | 0.271 | 0.0111 | N.D. | 0.047 | 0.036 | 1.809 |
|  | 75 | 29.8946 | 34.1892 | 6.2224 | 0.334 | 0.0168 | N.D. | 0.048 | 0.043 | 1.806 |
|  | 105 | 26.8769 | 34.7571 | 5.0264 | 0.359 | 0.0143 | 2.185 | 0.159 | 0.329 | 4.002 |
|  | 200 | 16.8275 | 35.1796 | 1.9832 | 0.039 | 0.2565 | 11.275 | 0.011 | 1.247 | 10.142 |
|  | 500 | 10.4456 | 34.9909 | 1.8071 | 0.001 | 0.0925 | 27.807 | 0.012 | 2.031 | 29.732 |
|  | 853 | 7.3301 | 34.9635 | 1.5323 | 0.000 | 0.0216 | 32.05 | 0.007 | 2.495 | 57.086 |
|  | 1000 | 6.5865 | 34.9327 | 1.7673 | 0.000 | 0.1586 | 34.86 | 0.009 | 2.697 | 79.958 |
|  | 2000 | 2.4434 | 34.7638 | 4.1907 | 0.000 | 0.0056 | 33.144 | 0.005 | 2.592 | 122.122 |
|  | 3000 | 1.4633 | 34.7256 | 5.081 | 0.000 | 0.0169 | 30.488 | 0.015 | 2.492 | 131.417 |
|  | 4000 | 0.8531 | 34.7088 | 6.0528 | 0.000 | 0.0165 | 29.708 | 0.009 | 2.331 | 129.725 |
|  | 4580 | 0.8104 | 34.7079 | 6.1549 | 0.000 | 0.0185 | 30.447 | 0.004 | 2.339 | 132.550 |
| E87-13 | 5 | 30.2533 | 34.5309 | 6.0181 | 0.082 | N.D. | 0.037 | 0.040 | 0.052 | 1.585 |
|  | 25 | 30.1905 | 34.5266 | 6.1605 | 0.125 | N.D. | 0.045 | 0.040 | 0.050 | 1.425 |
|  | 50 | 29.7821 | 34.6867 | 6.2321 | 0.195 | 0.032 | 0.035 | 0.040 | 0.054 | 1.552 |
|  | 75 | 27.0118 | 34.9076 | 5.0116 | 0.480 | 0.011 | 3.143 | 0.118 | 0.346 | 3.975 |
|  | 96 | 22.7033 | 35.2212 | 3.6537 | 0.423 | 0.058 | 8.831 | 0.214 | 0.911 | 14.638 |
|  | 200 | 14.379 | 35.1363 | 2.1319 | 0.008 | N.D. | 22.894 | 0.014 | 1.710 | 24.813 |
|  | 645 | 8.9753 | 35.0206 | 1.3234 | 0.000 | 0.027 | 34.882 | 0.012 | 2.571 | 54.200 |
|  | 1000 | 6.6925 | 34.9399 | 1.6466 | 0.000 | N.D. | 36.72 | 0.011 | 2.842 | 80.871 |
|  | 2000 | 2.4602 | 34.7649 | 4.1993 | 0.000 | N.D. | 35.343 | 0.009 | 2.743 | 120.597 |
|  | 3000 | 1.5223 | 34.7289 | 5.0438 | 0.000 | N.D. | 34.44 | 0.011 | 2.695 | 128.173 |
|  | 4000 | 1.0758 | 34.7154 | 5.5489 | 0.000 | 0.020 | 33.723 | 0.010 | 2.628 | 129.382 |
| EQ-1 | 5 | 29.9784 | 34.588 | 6.1602 | 0.221 | 0.0198 | N.D. | 0.045 | 0.072 | 1.845 |
|  | 25 | 29.9966 | 34.6019 | 6.1597 | 0.217 | 0.0236 | N.D. | 0.047 | 0.052 | 1.813 |
|  | 79 | 27.3182 | 35.0395 | 5.541 | 0.469 | 0.1322 | 1.813 | 0.142 | 0.300 | 3.037 |
|  | 100 | 25.6404 | 35.306 | 5.1691 | 0.315 | 0.0579 | 1.589 | 0.638 | 0.439 | 3.798 |
|  | 200 | 13.8109 | 35.1361 | 2.1962 | 0.004 | 0.0968 | 20.891 | 0.007 | 1.623 | 18.394 |
|  | 500 | 9.8569 | 34.9891 | 1.6772 | 0.000 | 0.6335 | 28.039 | 0.003 | 2.209 | 35.661 |
|  | 766 | 8.2017 | 34.9978 | 1.3708 | 0.000 | 0.0501 | 33.295 | 0.017 | 2.659 | 61.453 |
|  | 1000 | 6.5109 | 34.9376 | 1.6753 | 0.000 | 0.1952 | 34.238 | 0.004 | 2.729 | 74.183 |
|  | 2000 | 2.6271 | 34.7704 | 4.0651 | 0.000 | 0.0461 | 32.528 | 0.003 | 2.607 | 117.694 |
|  | 3000 | 1.4805 | 34.7259 | 5.0575 | 0.000 | 0.0330 | 31.997 | 0.004 | 2.581 | 127.930 |
|  | 4000 | 1.0764 | 34.7136 | 5.4889 | 0.000 | 0.0551 | 31.646 | 0.002 | 2.534 | 130.839 |
|  | 4650 | 1.035 | 34.713 | 5.6311 | 0.000 | 0.0485 | 30.827 | 0.005 | 2.416 | 127.509 |
| H14 | 5 | 29.7288 | 33.807 | 6.1484 | 0.153 | 0.060 | 0.111 | 0.039 | 0.087 | 1.771 |
|  | 25 | 29.7405 | 33.8062 | 6.1628 | 0.158 | 0.070 | 0.103 | 0.037 | 0.084 | 1.812 |
|  | 60 | 29.2904 | 34.2828 | 5.4794 | 0.643 | 0.123 | 0.299 | 0.034 | 0.112 | 0.997 |
|  | 100 | 21.1147 | 34.6198 | 2.1117 | 0.069 | 0.087 | 7.119 | 0.010 | 1.032 | 7.192 |
|  | 200 | 13.571 | 35.0251 | 0.7119 | 0.001 | 0.053 | 27.58 | 0.011 | 2.188 | 28.065 |
|  | 500 | 10.1485 | 35.048 | 0.9217 | 0.001 | 0.100 | 32.616 | 0.007 | 2.506 | 41.575 |
|  | 793 | 8.1385 | 34.9927 | 0.9666 | 0.000 | 0.325 | 36.383 | 0.019 | 2.918 | 67.689 |
|  | 1000 | 6.7939 | 34.9331 | 1.6076 | 0.000 | 0.025 | 37.752 | 0.006 | 2.978 | 84.305 |
|  | 2000 | 2.743 | 34.7779 | 3.7516 | 0.000 | 0.016 | 35.331 | 0.005 | 2.815 | 128.908 |
|  | 3200 | 1.341 | 34.7212 | 5.1008 | 0.000 | 0.013 | 33.556 | 0.005 | 2.660 | 137.653 |
| H4 | 5 | 29.7454 | 34.1031 | 6.1086 | 0.227 | 0.026 | 0.079 | 0.053 | 0.032 | 2.261 |
|  | 25 | 29.7432 | 34.1012 | 6.1316 | 0.249 | 0.041 | 0.067 | 0.031 | 0.028 | 1.470 |
|  | 69 | 28.5703 | 34.4902 | 5.2878 | 0.606 | 0.029 | 2.1 | 0.454 | 0.254 | 2.669 |
|  | 100 | 20.7211 | 34.782 | 1.3436 | 0.063 | 0.047 | 15.922 | 0.019 | 1.618 | 17.301 |
|  | 150 | 15.5057 | 34.9374 | 0.5939 | 0.011 | 0.021 | 20.711 | 0.009 | 1.958 | 24.997 |
|  | 200 | 13.1096 | 35.0414 | 0.446 | 0.003 | 0.049 | 25.77 | 0.004 | 2.070 | 27.150 |
|  | 418 | 10.7452 | 35.0421 | 1.3275 | 0.002 | 0.011 | 30.528 | 0.007 | 2.280 | 31.169 |
|  | 500 | 10.3199 | 35.0407 | 1.2033 | 0.001 | 0.190 | 31.528 | 0.006 | 2.340 | 34.456 |
|  | 1000 | 6.4518 | 34.9244 | 1.7077 | 0.000 | 0.009 | 37.119 | 0.005 | 2.925 | 87.183 |
| E87-21 | 5 | 30.7996 | 34.57 | 6.1543 | 0.080 | 0.082 | 0.065 | 0.040 | 0.030 | 1.704 |
|  | 25 | 30.5597 | 34.5795 | 6.1934 | 0.077 | 0.035 | 0.083 | 0.039 | 0.033 | 2.259 |
|  | 50 | 30.1201 | 34.6966 | 6.2856 | 0.138 | N.D. | 0.108 | 0.041 | 0.045 | 2.705 |
|  | 94 | 28.9771 | 34.6269 | 5.895 | 0.770 | N.D. | 1.765 | 0.058 | 0.298 | 9.505 |
|  | 100 | 28.561 | 34.6332 | 5.4555 | 0.626 | 0.011 | 2.88 | 0.386 | 0.398 | 10.293 |
|  | 150 | 19.616 | 34.8033 | 1.4541 | 0.098 | 0.016 | 20.757 | 0.044 | 1.688 | 25.965 |
|  | 200 | 15.467 | 34.9729 | 0.6066 | 0.004 | N.D. | 28.471 | 0.019 | 2.153 | 32.407 |
|  | 420 | 10.8626 | 35.0543 | 0.2437 | 0.003 | 0.087 | 34.22 | 0.011 | 2.496 | 43.067 |
|  | 1000 | 6.5263 | 34.9298 | 1.5184 | 0.000 | 0.012 | 37.469 | 0.018 | 2.891 | 84.707 |
|  | 2000 | 2.582 | 34.7725 | 3.7931 | 0.000 | 0.044 | 34.177 | 0.010 | 2.746 | 125.142 |
|  | 3000 | 1.5154 | 34.7273 | 4.8554 | 0.000 | 0.108 | N.D. | N.D. | N.D. | N.D. |
|  | 4050 | 1.096 | 34.7146 | 5.227 | 0.000 | 0.179 | 33.551 | 0.012 | 2.681 | 140.142 |
| F5 | 5 | 30.4263 | 33.676 | 6.1633 | 0.072 | 0.024 | 0.074 | 0.043 | 0.046 | 1.207 |
|  | 25 | 30.3026 | 34.684 | 6.3091 | 0.104 | 0.021 | 0.049 | 0.037 | 0.048 | 1.115 |
|  | 50 | 29.6466 | 34.9225 | 6.2995 | 0.262 | 0.012 | 0.062 | 0.042 | 0.053 | 1.224 |
|  | 70 | 28.1231 | 35.1005 | 5.0321 | 0.643 | 0.020 | 2.397 | 0.241 | 0.323 | 4.543 |
|  | 100 | 24.3869 | 34.8019 | 3.0252 | 0.161 | 0.018 | 8.979 | 0.028 | 0.897 | 10.604 |
|  | 150 | 16.5697 | 34.8972 | 0.8519 | 0.023 | 0.103 | 18.26 | 0.588 | 1.206 | 19.887 |
|  | 200 | 13.7103 | 35.0069 | 0.4827 | 0.004 | 0.082 | 28.313 | 0.361 | 1.841 | 26.169 |
|  | 576 | 9.6542 | 35.0393 | 0.5849 | 0.000 | 0.016 | 37.73 | 0.009 | 2.575 | 50.155 |
|  | 1000 | 6.657 | 34.931 | 1.5746 | 0.000 | 0.026 | 38.536 | 0.003 | 2.736 | 81.569 |
|  | 2000 | 2.6258 | 34.7739 | 3.8775 | N.D. | 0.025 | 37.731 | 0.008 | 2.663 | 121.939 |
| E87-25 | 5 | 30.9867 | 32.8554 | 6.2771 | 0.175 | N.D. | N.D. | 0.046 | 0.059 | 2.621 |
|  | 25 | 29.8348 | 33.1016 | 6.4566 | 0.260 | 0.042 | N.D. | 0.046 | 0.050 | 2.988 |
|  | 50 | 28.1531 | 33.9911 | 6.3189 | 0.429 | 0.015 | N.D. | 0.048 | 0.081 | 2.446 |
|  | 69 | 27.7203 | 34.0979 | 5.2816 | 0.542 | N.D. | 1.409 | 0.075 | 0.153 | 3.856 |
|  | 100 | 23.8475 | 34.8066 | 1.1989 | 0.243 | N.D. | 17.039 | 0.056 | 1.370 | 13.687 |
|  | 150 | 18.7619 | 34.89 | 0.7709 | 0.013 | 0.018 | 24.458 | 0.019 | 1.812 | 23.042 |
|  | 230 | 13.4681 | 34.9934 | 0.3294 | 0.004 | 0.029 | 31.709 | 0.007 | 2.330 | 32.105 |
|  | 500 | 9.743 | 35.0209 | 0.4383 | 0.001 | 0.031 | 36.936 | 0.003 | 2.578 | 48.963 |
|  | 1000 | 6.7496 | 34.9356 | 1.3457 | 0.000 | 0.044 | 38.585 | 0.004 | 2.821 | 77.629 |
|  | 2000 | 2.6443 | 34.774 | 3.6641 | 0.000 | 0.016 | 37.054 | 0.002 | 2.758 | 123.980 |
|  | 3000 | 1.5291 | 34.7285 | 4.6627 | 0.000 | 0.194 | 36.464 | 0.008 | 2.638 | 131.076 |
|  | 3650 | 1.1957 | 34.7169 | 4.7179 | 0.000 | 0.024 | 37.033 | 0.010 | 2.623 | 142.048 |
| E87-31 | 5 | 31.6213 | 31.8852 | 6.086 | 0.070 | 0.022 | N.D. | 0.041 | N.D. | N.D. |
|  | 25 | 30.6104 | 32.0274 | 6.4682 | 0.109 | N.D. | N.D. | 0.040 | N.D. | N.D. |
|  | 50 | 28.4782 | 33.3327 | 6.0701 | 0.369 | 0.010 | N.D. | 0.042 | N.D. | N.D. |
|  | 75 | 27.2090 | 33.8938 | 3.3713 | 0.402 | 0.036 | 4.117 | 0.457 | 0.610 | 7.449 |
|  | 100 | 24.6813 | 34.377 | 0.4862 | 0.203 | 0.013 | 15.24 | 0.105 | 1.286 | 12.263 |
|  | 165 | 16.4481 | 34.8841 | 0.042 | 0.041 | N.D. | 25.99 | 0.058 | 2.143 | 28.584 |
|  | 200 | 14.3794 | 34.9481 | 0.00 | 0.019 | N.D. | 28.919 | 0.014 | 2.315 | 32.204 |
|  | 500 | 10.0049 | 35.0223 | 0.00 | 0.009 | N.D. | 36.25 | 0.003 | 2.679 | 48.416 |
|  | 1000 | 6.598 | 34.9279 | 1.2178 | 0.001 | N.D. | 37.657 | 0.002 | 2.897 | 85.600 |
|  | 2000 | 2.5637 | 34.7699 | 3.5194 | 0.000 | 0.026 | 36.119 | 0.003 | 2.810 | 130.006 |
|  | 2890 | 1.5318 | 34.7285 | 4.4597 | 0.000 | 0.015 | 34.613 | 0.006 | 2.584 | 144.441 |

^*^N.D., no data. T: Temperature, DO: dissolved oxygen, Chl *a*: chlorophyll *a*, DSi: dissolved silicon, DIP: dissolved inorganic phosphorus.

**Table. S2 Network topological features of FL and PA communities**

|  | FL | PA |
| --- | --- | --- |
| Nodes | 395 | 235 |
| Edges | 1397 | 895 |
| Average Clustering Coefficient | 0.568 | 0.575 |
| Average Degree | 7.073 | 7.617 |
| Graph Density | 0.018 | 0.033 |
| Average Path Length | 3.244 | 3.789 |
| Network Diameter | 12 | 13 |
| Positive Nodes | 99.86% | 97.88% |
| Negative Nodes | 0.14% | 2.12% |
